# Single-Atom Catalysts through Pressure-Controlled Metal Diffusion

**DOI:** 10.1021/jacs.4c03066

**Published:** 2024-07-11

**Authors:** Samir H. Al-Hilfi, Xikai Jiang, Julian Heuer, Srinu Akula, Kaido Tammeveski, Guoqing Hu, Juan Yang, Hai. I. Wang, Mischa Bonn, Katharina Landfester, Klaus Müllen, Yazhou Zhou

**Affiliations:** † School of Materials Science and Engineering, Jiangsu University, Zhenjiang 212013, Jiangsu, China; ‡ Max Planck Institute for Polymer Research, 55128 Mainz, Germany; § State Key Laboratory of Nonlinear Mechanics, Institute of Mechanics, Chinese Academy of Science, Beijing 100190, China; ∥ Institute of Chemistry, University of Tartu, Ravila 14a, 50411 Tartu, Estonia; ⊥ Department of Engineering Mechanics, State Key Laboratory of Fluid Power and Mechatronic Systems, Zhejiang University, Hangzhou 310027, Zhejiang, China; # Nanophotonics, Debye Institute for Nanomaterials Science, Utrecht University, Princetonplein 1, 3584 CC Utrecht, The Netherlands

## Abstract

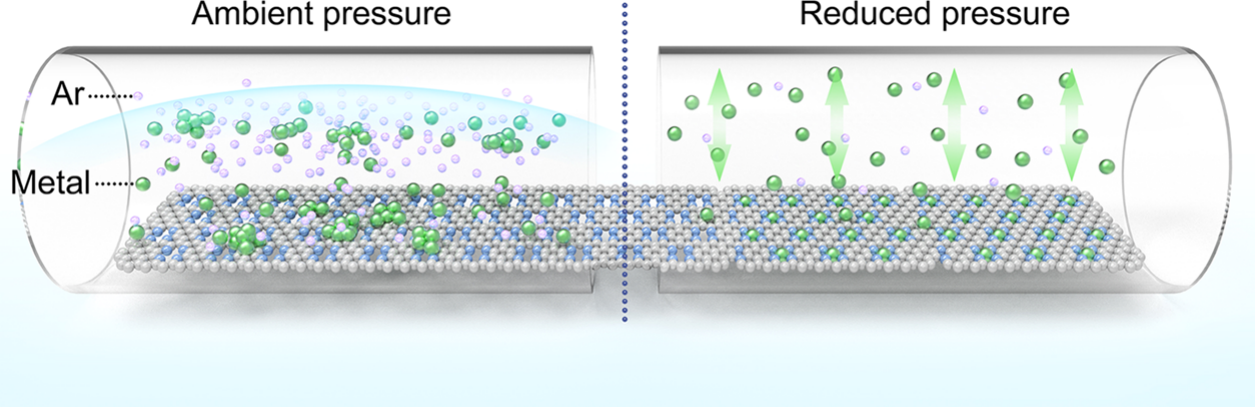

Single-atom catalysts (SACs) open up new possibilities for advanced technologies. However, a major complication in preparing high-density single-atom sites is the aggregation of single atoms into clusters. This complication stems from the delicate balance between the diffusion and stabilization of metal atoms during pyrolysis. Here, we present pressure-controlled metal diffusion as a new concept for fabricating ultra-high-density SACs. Reducing the pressure inhibits aggregation substantially, resulting in almost three times higher single-atom loadings than those obtained at ambient pressure. Molecular dynamics and computational fluid dynamics simulations reveal the role of a metal hopping mechanism, maximizing the metal atom distribution through an increased probability of metal–ligand binding. The investigation of the active site density by electrocatalytic oxygen reduction validates the robustness of our approach. The first realization of Ullmann-type carbon–oxygen couplings catalyzed on single Cu sites demonstrates further options for efficient heterogeneous catalysis.

## 1 Introduction

Heterogeneous catalysis plays an indispensable tool in modern industrial processes.^1^ When supported nanoparticles are downsized to the single-atom limit, the resulting single-atom catalysts (SACs) have proven especially valuable.^2−4^ These individually dispersed metal sites possess defined coordination structures, enabling reaction pathways that differ from those of traditional heterogeneous catalysis. For instance, our group has recently reported carbon–carbon bond formation by a heterogeneous single-atom Pt-catalyzed Heck reaction.^5^ While offering high stability even in harsh environments and maximum utilization of catalytic metals, SACs also satisfy the key goals of sustainable chemistry.^6−9^ Nevertheless, limited reproducibility and scalability of SAC preparation hinder their further practical applications, and efficient methods of SAC synthesis are urgently needed.^10,11^

**Figure 1:**
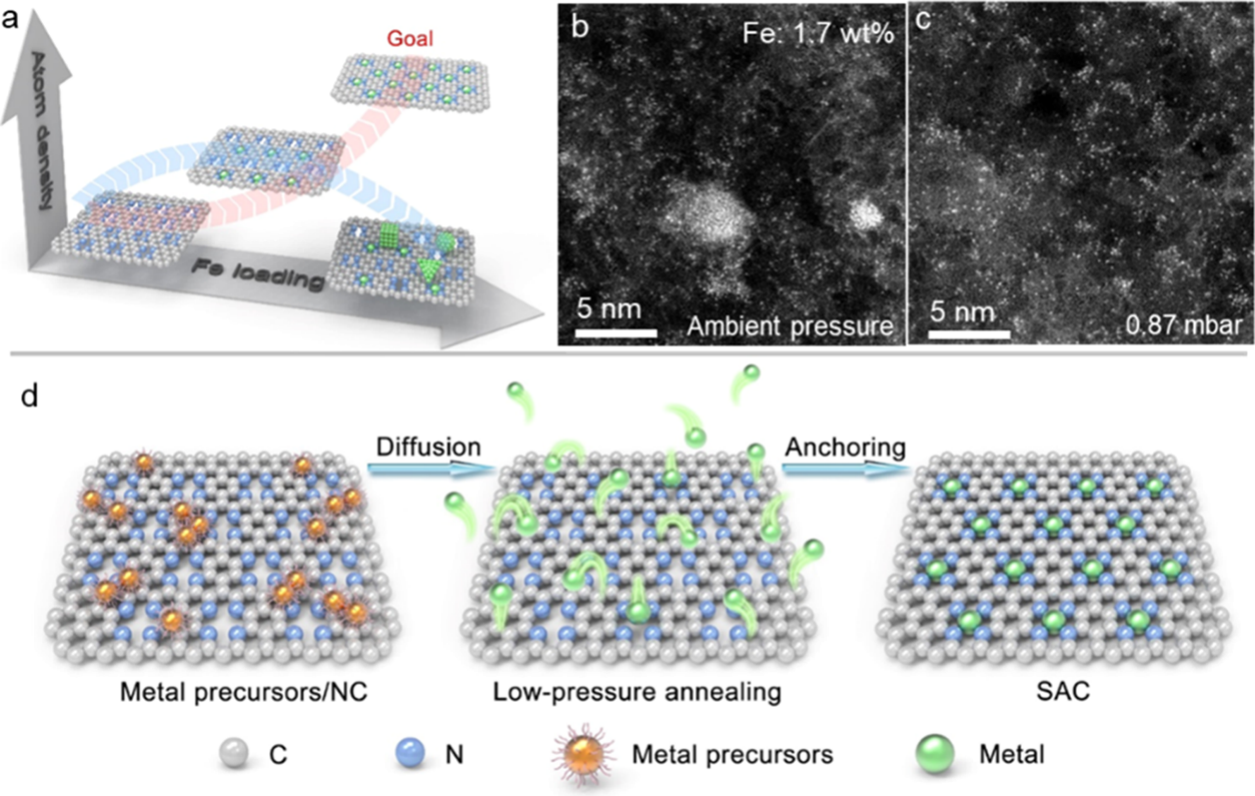
Synthesis of high-density SACs. (a) Increasing the site density of single atoms with increased metal loading upon pyrolysis under ambient pressure. (b) Comparison of the morphologies of Fe-NC materials prepared by pyrolyzing NaFe-EDTA/NC under (b) ambient pressure and (c) 0.87 mbar. (d) Schematic illustration of the pressure-controlled metal diffusion approach for preparing SACs.

Numerous approaches have been reported for fabricating SACs.^12^ These include atomic layer deposition (ALD),^13^ chemical vapor deposition (CVD),^14^ electrochemical,^15^ and mechanochemical protocols.^16^ The pyrolysis of mixed support materials and metal precursors has been proven to be the most suitable method for large-scale production.^17−20^ However, during the necessary annealing process, undesirable clusters are often formed due to the unavoidable aggregation of metal atoms (Figure 1a,b), limiting final single-atom contents to 2 wt %. To increase the atomic load, various concepts have been employed, including encapsulation into metal–organic frameworks, covalent organic frameworks, or polymers,^21−26^ with simultaneous coordination site design.^27−29^ The complex wet-chemical steps and expensive organic precursors significantly reduce the scalability and increase costs. One anticipates that the synthesis of dense SAC depends sensitively upon metal diffusion during pyrolysis. Indeed, diffusion control constitutes a critical step in deposition methods like physical vapor deposition and CVD.^30^ In principle, working at low pressure (e.g., high vacuum) is expected to reduce collisions between reactant molecules and achieve a homogeneous distribution of precise nanostructures on substrates. We reasoned that similarly, pressure control might help increase the density of SACs on the support.

We realized this concept by employing nitrogen-doped graphitic carbon (NC) with high thermodynamical stability as the support and low pressures during pyrolysis (Figure 1c). A wide range of SACs, including both nonprecious and precious metals with single-atom loadings nearly three times higher than that obtained at ambient pressure, has been achieved. Molecular dynamics (MD) and computational fluid dynamics (CFD) simulations demonstrate that reduced pressure significantly enhances metal diffusion due to the high mean free path of the atoms and thus minimizes agglomeration. The prepared SACs furnish high performance in an electrocatalytic oxygen reduction reaction (ORR) and high yields in Ullmann C–O coupling.

## 2 Results and Discussion

### 2.1 Preparation and Structural Characterization of SACs

The pyrolysis process is often carried out at high temperatures to activate metal precursors into single atoms, especially for nonprecious metals (>700 °C). For the synthesis of Fe SACs through controlled pressure pyrolysis, sodium ferric ethylenediaminetetraacetate (NaFe-EDTA) was mixed with porous NC in methanol by sonication and stirring (Figure 1d and see details in Section 4). The mixture was then thermally activated at 900 °C in an argon (Ar) atmosphere under low pressure. The resulting catalysts are denoted as Fe_*x*_-NC^*y*^, where *x* refers to the mass ratio of NaFe-EDTA to NC and *y* represents the pyrolysis pressure.

**Figure 2:**
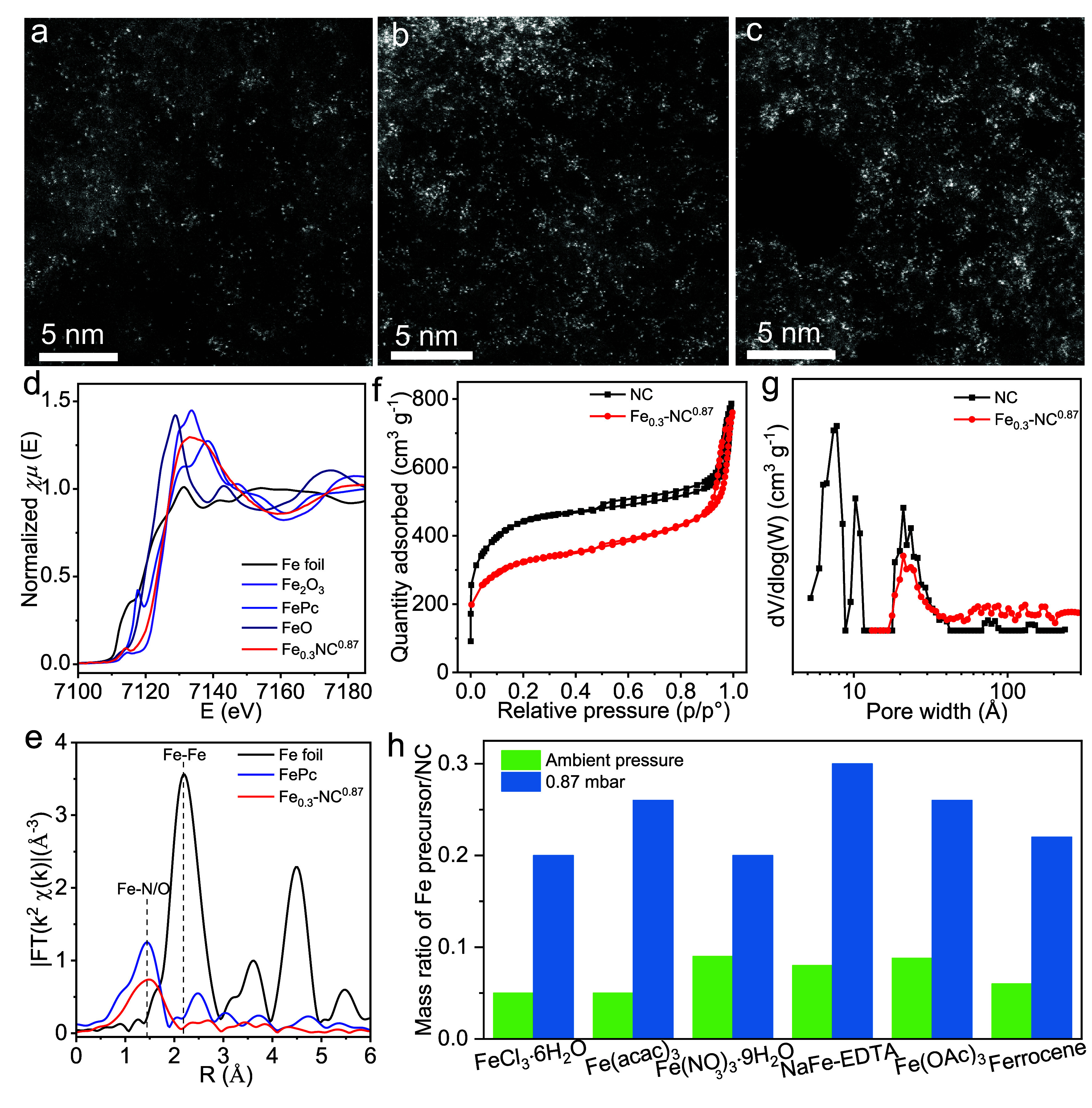
Characterization of Fe-NC catalysts synthesized at a pressure of 0.87 mbar. AC-HAADF-STEM images of (a) Fe_0.1_-NC^0.87^, (b) Fe_0.2_-NC^0.87^, and (c) Fe_0.3_-NC^0.87^, (d) XANES spectra of Fe_0.3_-NC^0.87^ together with those of Fe foil, FeO, Fe_2_O_3_, and FePc standards, (e) Fourier-transform EXAFS spectra, (f) N_2_ adsorption/desorption isotherms of NC and Fe_0.3_-NC^0.87^, (g) the corresponding pore size distribution curves calculated from the adsorption branches via nonlocal density functional theory (NLDFT), and (h) the maximum amount of several Fe precursors on the NC support for Fe SAC synthesis at ambient pressure and 0.87 mbar, respectively.

Characterization by high-angle annular dark-field scanning transmission electron microscopy (HAADF-STEM) showed that Fe clusters and nanoparticles were formed in all materials prepared at ambient pressure (Fe-NC^AP^), except for the Fe_0.08_-NC^AP^ with a low Fe loading of 1.4 wt % (Supporting Figure 1). Switching to reduced pressure suppressed the previously observed Fe clustering at comparable Fe loadings, resulting in a significantly improved Fe atom distribution (Supporting Figure 2). The pressure was varied from ambient pressure (AP) to 1.4, 0.87, 0.52, and 0.14 mbar. Remarkably, at reduced pressure, the coverage of Fe atoms on the surface of the NC support could be adjusted by the amount of Fe precursor (shown exemplarily for 0.87 mbar in Figure 2a–c; see also Supporting Figures 3–6). The highest loading of single Fe atoms (∼4.5 wt %) was achieved in Fe_0.3_-NC^0.87^, which was 3.2 times higher than at ambient pressure, according to inductively coupled plasma atomic emission spectroscopy (ICP-AES) analysis (Supporting Table 1).

Single atoms were examined using X-ray photoelectron spectroscopy (XPS), synchrotron radiation-based X-ray absorption near-edge structure (XANES), and extended X-ray absorption fine structure (EXAFS). Compared to the NC support, the high-resolution XPS spectrum of N 1s in Fe_0.3_-NC^0.87^ displayed an emerging peak at 399.5 eV, which could be assigned to the metal–N moiety (Supporting Figure 7a).^31^ This indicates the anchoring of Fe atoms by the nitrogen sites. Fe 2p XPS and Fe K-edge XANES analysis confirmed a predominant Fe oxidation state of +3 (Figure 2d and Supporting Figure 7b). An investigation of the coordination environment of Fe atoms via EXAFS revealed a major peak at about 1.5 Å, corresponding to the Fe–N/O bond (Figure 2e and Supporting Figure 8a). No signatures of Fe–Fe coordination (>2.0 Å) were detected, verifying the absence of Fe aggregates. Best-fit EXAFS spectra indicated that a single Fe center was coordinated with four N atoms and two adsorbed O atoms, i.e., O_2_–Fe(III)-N_4_ in Fe_0.3_-NC^0.87^ (Supporting Figure 8b and Table 2).^32^

Micropores are known to be the primary sites for hosting single atoms on NC supports.^33^ The NC support exhibited the coexistence of ultramicropores (∼0.6 nm), micropores (∼1.0 nm), and mesopores (Figure 2g). In Fe_0.3_-NC^0.87^, these ultramicropores were absent, resulting in a lower Brunauer–Emmett–Teller surface area of 1168 m^2^ g^–1^ compared to 1499 m^2^ g^–1^ of the NC support (Supporting Table 3). In addition, the NC support and Fe_0.3_-NC^0.87^ exhibited nearly similar micropore and mesopore characteristics. These observations demonstrate that the ultramicropores are utilized to stabilize Fe atoms, enabling the high density (Figure 2d,e).^34,35^

**Figure 3:**
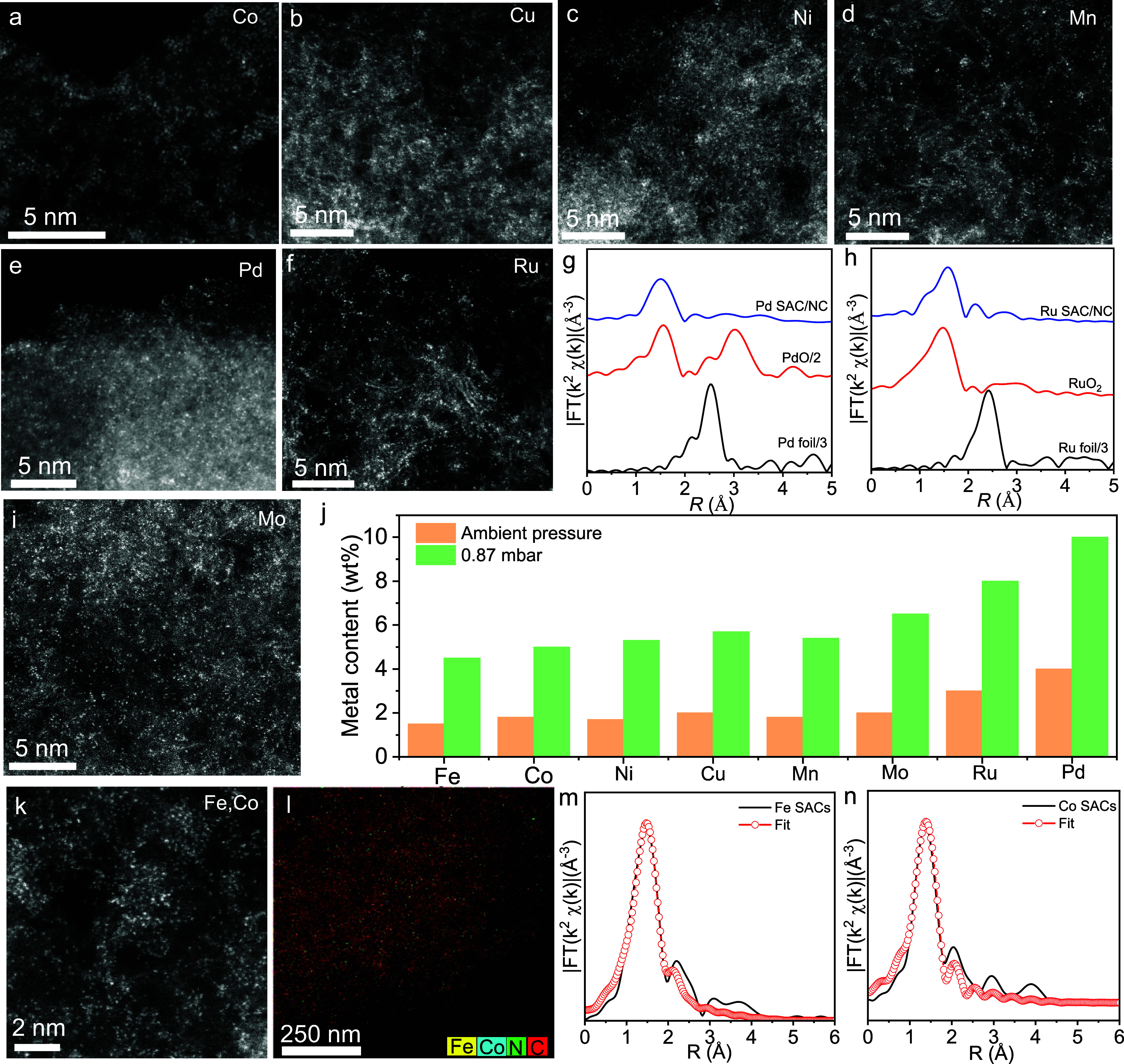
Synthesis and Characterization of other SACs. AC-HAADF-STEM images of SACs: (a) Co, (b) Cu, (c) Ni, (d) Mn, (e) Pd, (f) Ru, and (i) Mo. Fourier-transform EXAFS spectra of (g) Pd SAC and (h) Ru SAC. (j) Maximum loadings of single-atom metals achieved under ambient pressure and 0.87 mbar, respectively. Characterization of the Fe–Co NC material, (k) AC- HAADF-STEM image, (l) EDXS layered elemental mapping image of Fe, Co, N, and C. EXAFS fitting curves of (m) Fe K-edge and (n) Co K-edge.

The effect of the metal precursors was investigated using six different Fe starting compounds, including Fe ions and organometallic complexes. At ambient pressures, Fe particles were formed in all samples (Supporting Figure 9). In contrast, the materials prepared at a reduced pressure of 0.87 mbar showed identical morphologies but always without apparent particle formation. Remarkably, more than three times the amount of metal precursors were converted into SACs at 0.87 mbar compared to ambient pressure (Figure 2h). We further synthesized a family of SACs, including both nonprecious and noble metals (i.g., Co, Ni, Mn, Cu, Mo, Pd, and Ru; for details, see Section 4). Their morphologies demonstrate a high density of single atoms on the NC supports (Figure 3). EXAFS analysis confirmed that most of the Pd and Ru were in the atomic state (Figure 3g,h). According to ICP-AES results, a loading of single atoms up to 3.5 times higher can be achieved at 0.87 mbar compared to that under ambient pressure (Figure 3j and Supporting Figure 10). In the case of Pd, the value of single-atom loading exceeded 10 wt %. Dual-atom catalysts (DACs), as an extension of SACs, have garnered significant attention due to their remarkable catalytic performance.^36−38^ We, therefore, modified our method to synthesize DACs, i.e., Fe–Co and Fe–Cu DACs. Having first prepared Fe SACs, the secondary metal of Co or Cu was then introduced using the same process to result in high-density DACs (Figure 3k–n and Supporting Figure 11).

### 2.2 Mechanistic Modeling of Pressure-Controlled Metal Diffusion

**Figure 4:**
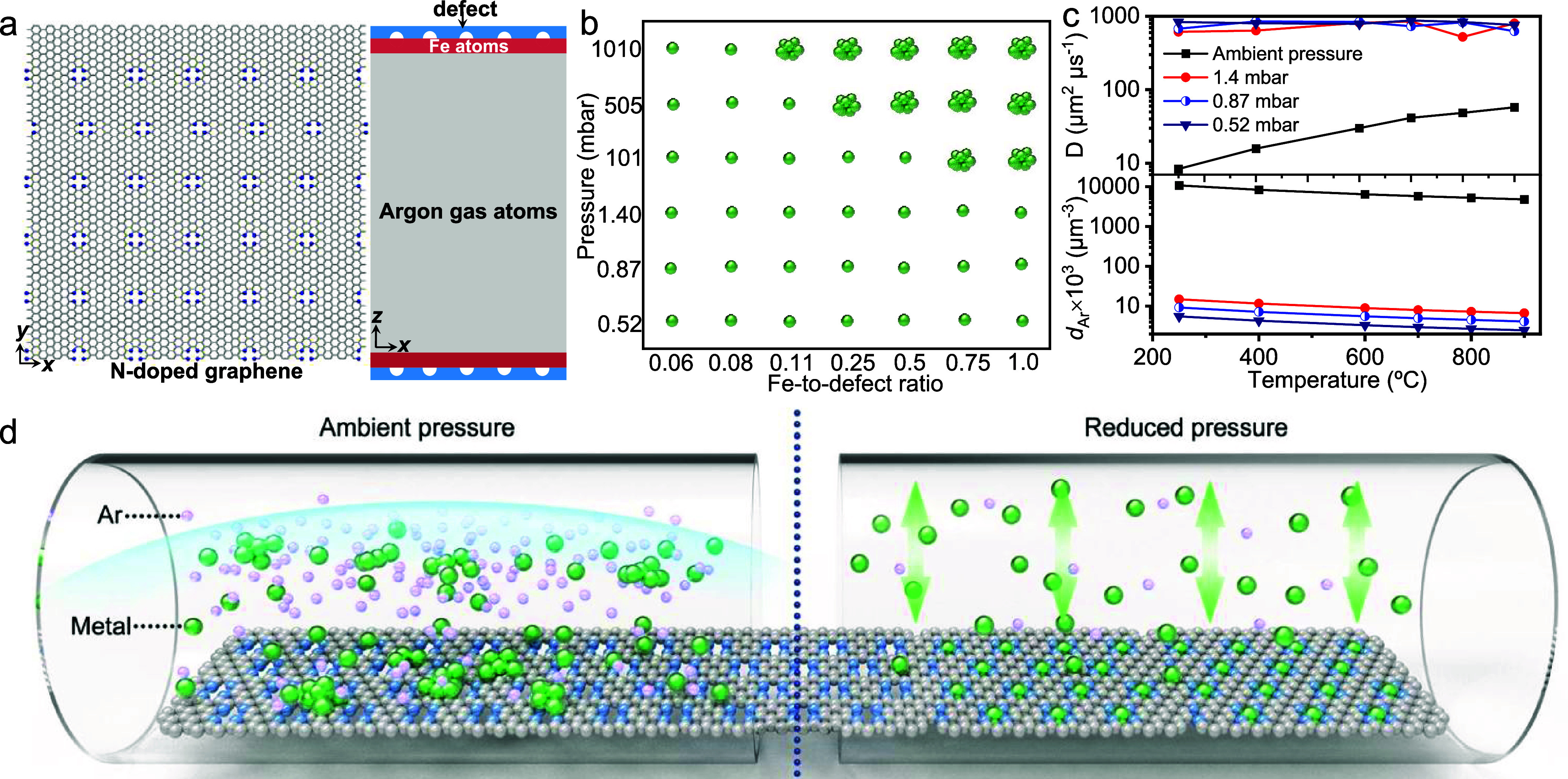
Mechanistic investigation of pressure-controlled metal diffusion. (a) Model of the MD simulation. Fe atoms near the NC support in the Ar atmosphere, (b) phase diagram for the formation of single atoms and clusters as a function of pressure and Fe-to-defect ratio. (c) Correlations of the Fe atom diffusion coefficient and Ar density with temperature and pressure. (d) Schematic of the proposed mechanism.

To gain a deeper understanding of the importance of pressure on single-atom formation, we employed MD modeling to investigate the evolution of metal atoms. Our model was constructed by using a graphene layer as the support. N-doping was introduced as an anchoring site (Figure 4a). The simulation started with the Fe atoms, assuming that NaFe-EDTA decomposition had released all of the Fe atoms. The phase diagram derived from the MD simulations clearly showed that lower pressure during the pyrolysis process favored the formation of single atoms on the NC support (Figure 4b) in accordance with experimental findings. We further recorded the evolution of Fe atoms with time (Videos), suggesting that metal atoms were exchanged between the gas phase and the graphene surface. Under ambient pressure (Video 1), Fe atoms tend to migrate on the surface of the support, leading to the formation of Fe clusters, unless the Fe density is reduced (e.g., Fe/defect ratio of 0.08) (Video 2). In contrast, at 0.87 mbar, Fe atoms initially diffuse upward rapidly and then bounce between the graphene sheets until captured on a N defect (Video 3). This significant difference in the diffusion behavior convincingly emphasizes the critical influence of pressure on SAC formation.

The diffusion coefficient (*D*) of the Fe atoms and Ar density (*d*_Ar_) at various pressures and temperatures was calculated, as shown in Figure 4c. Temperature-dependent diffusion of Fe atoms was observed at ambient pressure, with a low *D* (57.8 μm^2^ μs^–1^) at 900 °C. At pressures below 1.4 mbar, the diffusion of Fe atoms changed from temperature-dependent behavior to a dependence on Ar density. The Ar density at 0.87 mbar was calculated to be 4.1 × 10^3^ μm^–3^ at 900 °C, which was more than 3 orders of magnitude lower than that at 1 bar (ambient pressure, 4.8 × 10^6^ μm^–3^). In contrast, the value of *D* increased by ∼1 order of magnitude to 625 μm^2^ μs^–1^, indicating substantially enhanced diffusion. Considering the broad range of distances between the surfaces of carbon nanoparticles in the powders, we incorporated variations by setting the distance (*H*) between neighboring graphene sheets in our model to 300, 10, and 5 μm. The simulations revealed the formation of single atoms on the graphene surface under these conditions. At 0.87 mbar, there are almost no collisions between Fe and Ar atoms due to the high mean free path of the Ar atoms (∼930 μm), which is much larger than the distance between graphene sheets. The diffusivities of Fe atoms under different H values far exceeded those observed at ambient pressure (Supporting Figure 12).

In addition to MD, we further employed CFD to determine the effect of pressure reduction on metal diffusion toward the surface (see details of the simulations in the Supporting Information). The boundary layer thickness is the distance normal to the wall (support surface) to a point where the flow velocity has essentially reached the free-stream velocity.^39^ A thinner boundary layer typically indicates faster diffusion and interaction between the metal species in the fluid and the support material.^40^ Our analysis revealed that as the pressure decreased from ambient pressure to 0.87 mbar, the average boundary layer thickness decreased from 3.7 to 1.1 cm, respectively (Supporting Table 4). Combined MD and CFD calculations demonstrated that the improved diffusion resulting from pressure reduction allows metal atoms to diffuse onto the support surface and provides dense SACs via a hopping mechanism (Figure 4d).

### 2.3 Enhanced Catalytic Performance of SACs

**Figure 5:**
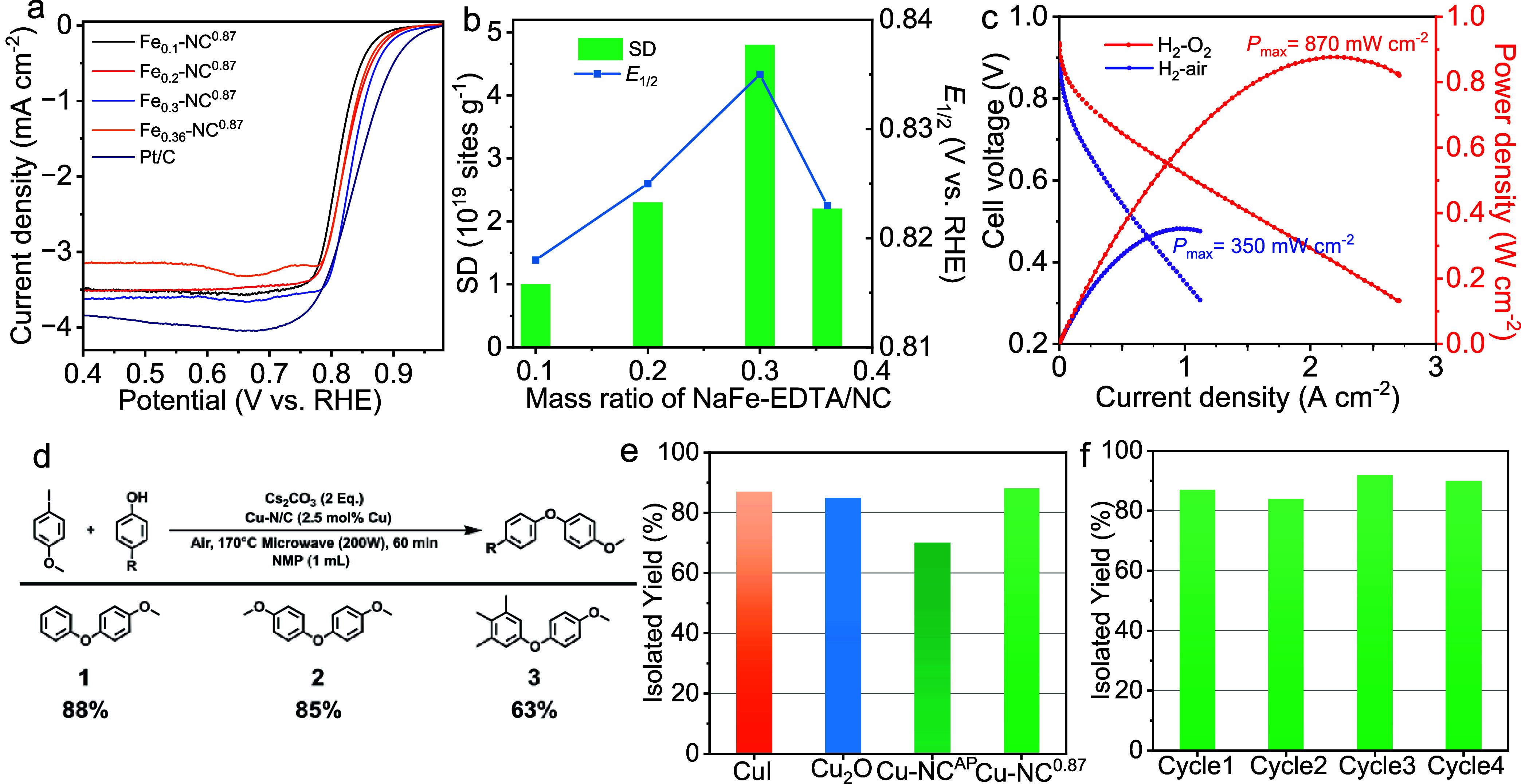
Catalytic performance of the SACs. (a-c) Electrocatalytic ORR performance. (a) ORR polarization curves of Pt/C andFe_*x*_-NC^0.87^ materials, measured in the O_2_-saturated 0.5 M H_2_SO_4_ at 5 mV s^–1^ with a rotation speed of 900 rpm. (b) Relationship between the site density, ORR activity, and the ratio of NaFe-EDTA/NC at 0.87 mbar. (c) H_2_–O_2_ and H_2_–air PEMFC polarization curves without *iR*-correction and power density curves. Cathode loading, 4.0 mg cm^–2^; anode loading, 0.2 mg_Pt_ cm^–2^; Nafion HP, H_2_/O_2_: 0.3/0.4 nlpm, H_2_/air: 0.3/0.6 nlpm, 100% relative humidity, 2.0 bar partial pressure, *T* = 80 °C. (d-f) Ullmann-type coupling. (d) Reaction scheme for investigation of SAC activity in Ullmann condensations. Iodoanisole (1 equiv), Cs_2_CO_3_ (2 equiv), and phenol derivative (1.5 equiv) were dissolved in NMP (1 mL) in a 10 mL pyrex tube equipped with a stir bar. The reaction mixture was submitted to a microwave synthesizer and irradiated to 170 °C for 1 h using 200 W. (e) Comparison of the catalytic activity of Cu-NC^0.87^ and Cu-NC^AP^, commercial CuI, and Cu_2_O at identical reaction conditions. (f) Recyclability performance of heterogeneous Cu-NC^0.87^ for the coupling of iodoanisole with phenol.

As a proof of concept, the catalytic ORR performance of the obtained Fe-NC materials was first evaluated by utilizing a rotating disk electrode (RDE) in an O_2_-saturated 0.5 M H_2_SO_4_ solution. As depicted in Supporting Figure 14, Fe_0.08_-NC^AP^ gave a good ORR activity with a half-wave potential (*E*_1/2_) of ∼0.81 V vs RHE. When the Fe loading increased, the ORR activity of Fe_0.2_-NC^AP^ (*E*_1/2_ = ∼ 0.79 V) decreased, indicating a low efficiency in the formation of active sites at ambient pressure. In contrast, reducing the pyrolysis pressure from 1000 to 0.87 mbar improved the activity substantially (Supporting Figure 15). Subsequently, no apparent change in activity was observed with a further reduction in the pressure. The optimal pressure was found to be 0.87 mbar. A further improvement in ORR activity was observed by increasing the Fe loading (Figure 5a). Specifically, the onset potential (*E*_onset_) increased from 0.88 V for Fe_0.1_-NC^0.87^ to 0.91 V for Fe_0.3_-NC^0.87^ at a loading of 0.6 mg of cm^–2^. The *E*_1/2_ increased from 0.82 to 0.84 V, only 10 mV less than that of a commercial Pt/C catalyst. Further increasing the loading of Fe resulted in a decrease in the activity of Fe_0.36_-NC^0.87^. We further examined the density of single Fe site (SD) by the *in situ* nitrite stripping method (Supporting Figures 16–18).^32,41^ The SD values were consistently higher for samples prepared at low pressure compared to ambient pressure (Supporting Table 5). The highest SD value was achieved for Fe_0.3_-NC^0.87^ (4.8 × 10^19^ sites g^–1^), with a 7.5-fold increase compared to Fe_0.1_-NC^AP^ (0.64 × 10^19^ sites g^–1^). In addition, Fe_0.3_-NC^0.87^ reached a high turnover frequency (TOF) of 1.2 e site^–1^ s^–1^ at 0.85 V, convincingly indicating high intrinsic activity (Supporting Figure 19). This value exceeded that of reported dense Fe-NC catalysts, including FeNC–CVD-750 (0.8 e site^–1^ s^–1^),^32^ ZIF-derived CNRS benchmark catalyst (0.14 e site^–1^ s^–1^),^42^ and Fe-NC^Δ−DCDA^ (0.13 e^–^ site^–1^ s^–1^).^43^ These analyses indicated that the enhancement of ORR activity with high loading of single Fe atoms is associated with (i) the accessibility of more active sites for overall catalysis^44^ and (ii) improved intrinsic activity of Fe sites due to the strong interactions between adjacent Fe atoms.^45^ To further evaluate the catalytic performance of Fe_0.3_-NC^0.87^ in practical applications, it was utilized as a cathode material in membrane electrode assemblies (MEAs) in fuel cells (Figure 5c). The catalyst generated a current density of 1.1 A cm^–2^ at 0.6 V, with an oxygen flow rate of 0.4 normal liters per minute (nlpm). The corresponding peak power density was 870 mW cm^–2^. Under H_2_–air conditions, a peak power density of 350 mW cm^–2^ was obtained for Fe_0.3_-NC^0.87^ at an airflow rate of 0.6 nlpm. The catalytic durability of Fe_0.3_-NC^0.87^ was tested using the RDE technique. By holding at a constant potential of 0.8 V for 60 h, a degradation in activity was observed. This could be associated with carbon corrosion and Fe aggregation (Supporting Figures 20 and 21).

### 2.4 Cu SAC-catalyzed Ullmann-type C–O Cross-coupling

Metal-catalyzed cross-coupling reactions belong to the most important protocols in organic chemistry.^46^ However, a transformation in cross-coupling reactions is required, transitioning from expensive noble metals (e.g., Pt or Pd)^47−49^ to more affordable and abundant alternatives (e.g., Cu).^50−53^ The Ullmann-type cross-coupling has emerged as particularly valuable due to its reliability, low toxicity, and remarkable versatility.^54^ The synthesis of biaryl ethers, which find extensive industrial application in pharmaceuticals and agrochemicals, is of special interest. Unfortunately, Ullmann-type couplings require high temperatures and catalyst loadings, leading to low atom efficiencies and large amounts of waste.^53^

Here, we present the first C–O bond formation via Ullmann condensation with heterogeneous Cu SACs (Figure 5d). The Cu-NC^0.87^ showed high catalytic activity with 88% isolated yield of the reaction between phenol and iodoanisole after 1 h at 170 °C using a microwave reactor. In contrast, Cu-NC^AP^ containing Cu single sites and nanoparticles exhibited only a 70% yield (Figure 5e and Supporting Figure 22). It follows that distributed single Cu atoms are more active than Cu nanoparticles. This conclusion is further supported by the fact that the majority of reported Cu-based nanoparticles (e.g., Cu, CuO, CuFe_2_O_4_) require extended reaction times (ranging from 3 to 24 h) and higher catalyst loadings (between 5 and 10 mol %) to achieve comparable results.^55^ To further validate the reactivity of Cu SACs, we investigated the effect of electron-poor and electron-rich phenol derivatives. Therefore, iodoanisole was coupled with 4-hydroxyanisole and 3,4,5-trimethylphenol under identical conditions. The products were obtained in 85% and 63% isolated yield, respectively. Next, the excellent recyclability of Cu-NC^0.87^ was demonstrated, maintaining high reactivity with 80–90% yield for the reaction of phenol with iodoanisole for up to 4 cycles. This is superior to many Cu-based heterogeneous catalysts, such as Cu NPs (63% yield, 12 h),^50^ Fe_3_O_4_@creatine-Cu(I) magnetic catalyst (75% yield, 4 cycles),^56^ and Cu_1.8_S nanoflowers/graphene oxide (55% yield, 3 cycles).^57^ STEM and AC-HAADF-STEM images illustrated a uniform distribution of dense Cu atoms and the absence of Cu aggregation in used Cu-NC^0.87^ (Supporting Figure 23). One concludes that the presented Cu SACs show promising applications for the Ullmann cross-couplings with excellent catalyst reactivity and stability.

## 3 Conclusions

We present a scalable and economically feasible approach to dense SACs by pressure-controlled metal diffusion during the pyrolysis process. Through a combination of empirical and theoretical evidence, the positive role of improved metal diffusion by a reduced pyrolysis pressure was demonstrated. The possible mechanism involved minimizing aggregation by reducing the concentration of metal atoms, thus maximizing the probability of metal binding to available anchoring sites under enhanced metal diffusion. The prepared Fe SAC and Cu SAC exhibit excellent performance in electrocatalytic ORR and C–O coupling reactions, respectively. The synthesis of a series of SACs, including both nonprecious and precious metals, demonstrates the robustness of our method. This work represents a critical step toward a scalable synthesis and widespread application of dense SACs and paves the way for the development of more economically feasible catalyst systems.

## 4 Methods

### 4.1 Preparation of Fe SACs under Low Pressure

The NC supports were prepared through a pyrolysis process involving ZIF-8 nanoparticles (50 nm) and NaCl at a mass ratio of 1.^58^ The pyrolysis was carried out at 910 °C under an Ar atmosphere for 2 h. To remove residual Zn, the NC supports were etched with 2 M HCl at 80 °C overnight, followed by washing with water and ethanol six times. In typical experiments, 50 mg of the NC powders was dispersed in 50 mL of methanol and sonicated for 2 h. Fifteen mg of NaFe-EDTA was then added, and the mixture was stirred continuously overnight. Methanol was then removed by using a rotary evaporator. The resulting mixture was heated to 900 °C (heating rate of 30 °C min^–1^) in a chemical vapor deposition system under a pressure of 0.87 mbar in an Ar atmosphere. After 1 h, the sample was obtained and was denoted Fe-NC.

### 4.2 Studying the Parameters

Fe-NC was selected as a representative example. The pressure was set at ambient pressure (AP), 1.4, 0.87, 0.52, and 0.14 mbar. The mass ratios between NaFe-EDTA and NC supports were 0.1, 0.2, 0.3, and 0.36. The obtained sample was labeled Fe_*x*_-NC^*y*^, where *x* refers to the mass ratio and *y* represents the pyrolysis pressure. To investigate the independence of the pressure-controlled metal diffusion approach on metal precursors, several other Fe precursors were utilized, including FeCl_3_·6H_2_O, Fe(acac)_3_, Fe(NO_3_)_3_·9H_2_O, Fe(OAc)_3_, and ferrocene. Their corresponding mass ratios between Fe precursor and NC were 0.2, 0.26, 0.2, 0.26, and 0.22.

### 4.3 Preparation of Other M SACs under Low Pressure

The M-NC (M = Co, Ni, Cu, Mn, Mo, Pd, and Ru) were synthesized under a pressure of 0.87 mbar using procedures similar to those used for Fe-NC. In each case, the corresponding metal precursors, namely, Co(acac)_3_, (15 mg), Ni(acac)_2_ (10.5 mg), Cu(acac)_2_, (10.5 mg), Mn(acac)_2_ (10.5 mg), MoCl_5_ (8 mg), Pd(acac)_2_ (12 mg), and Ru(acac)_3_ (12 mg), were mixed with 50 mg NC supports. For the Cu, the thermal reduction process was carried out at 700 °C. For Pd and Ru, the annealing process was performed in a H_2_/Ar environment (v/v: 1/10) at 200 °C for 1 h.

### 4.4 Preparation of Fe,Co and Fe,Cu DACs

First, Fe_0.2_-NC^0.87^ was synthesized. 50 mg of Fe-NC was dispersed in 50 mL of methanol through sonication for 1 h. To introduce Co or Cu into Fe-NC, a methanol solution (10 mL) containing 5 mg of Co(acac)_3_ or 5 mg of Cu(acac)_2_ was added into Fe_0.2_-NC^0.87^ solution with continuous stirring for 2 h. The methanol was then removed by rotary evaporation. The resulting powders were thermally treated under 0.87 mbar in an Ar atmosphere. The Fe,Co-NC and Fe,Cu-NC were obtained at 900 and 700 °C, respectively.

### 4.5 Physical Characterization

Morphological analysis was performed using TEM, and elemental mapping images along with EDXS were acquired on a Tecnai G2 F30 S-Twin (FEI, Netherlands) instrument. HAADF-STEM images were collected using a Theims Z field emission electron microscope (FEI, The Netherlands) working at 200 kV. X-ray diffraction (XRD) measurements were carried out on a Rigaku SmartLab diffractometer with Cu Kα X-rays (λ = 1.5406 Å) and a scanning speed of 0.4° min^–1^. XPS experiments were conducted on an Axis Ultra DLD imaging XPS using hybrid mode (700 μm × 300 μm) with an 80 eV pass energy for survey spectra and 20 eV pass energy for high-resolution spectra of elements. The Fe contents in the samples were tested by ICP-AES on a VISTA MPX instrument (Varian Inc.). Specific surface area and pore size distribution were measured by N_2_ physisorption analysis on a Quantachrome SI-MP Instrument at 77 K. The degassing process was performed at 120 °C for 8 h under a vacuum with a continuous N_2_ flow before measurement. The pore size distribution curves calculated from the adsorption branches via NLDFT, and XAS experiments were carried out at the BL14W1 station of the Shanghai Synchrotron Radiation Facility (SSRF), which operated at 2.5 GeV with a maximum current of 250 mA. Data reduction, analysis, and EXAFS fitting were performed using the ATHENA module implemented in the IFEFFIT software packages according to standard procedures.

### 4.6 MD Simulations

MD simulations were performed using the LAMMPS package to study the dynamics of Fe and Ar atoms near the NC surface.^59^ The simulation system consisted of Ar atoms sandwiched by two planar walls, with each wall modeled as the NC. The walls were placed perpendicular to the *z*-direction, with a distance of 10 μm between the two walls, ensuring that the Ar was bulk-like. A periodic boundary condition was applied along the *x*- and *y*-directions, with simulation system lengths of 10.21 and 10.07 nm, respectively. Under different pressures, the number of Ar atoms in the system was calculated using the ideal gas law, with the Ar atoms distributed randomly between the two walls using Packmol.^60^ The Ar number density was calculated by dividing the total number of Ar atoms in the simulation system by the total volume of the system. In the initial configurations, Fe atoms were uniformly placed in a plane 1 nm from the wall. The number of Fe atoms varied to produce different loadings of Fe atoms. C–Fe, C–Ar, N–Fe, N–Ar, and Fe–Ar interactions were modeled by using the Lennard-Jones (LJ) potential. The LJ parameters were σ_C–Ar_ = 0.34025 nm, ϵ_C–Ar_ = 0.00621 kcal mol^–1^; σ_C–Fe_ = 0.385 nm, ϵ_C–Fe_ = 0.03105 kcal mol^–1^; σ_Ar–Ar_ = 0.3405 nm, ϵ_Ar-Ar_ = 0.01034 kcal mol^–1^; σ_Ar–Fe_ = 0.38525 nm, ϵ_Ar–Fe_ = 0.0517 kcal mol^–1^; σ_Ar–N_ = 0.33275 nm, ϵ_Ar–N_ = 0.008731 kcal mol^–1^.^61,62^ The cutoff radius for LJ interactions was 1.2 nm. Fe–Fe and Fe–N interactions were modeled using the MEAM potential.^63,64^ A Nose–Hoover thermostat controlled the temperature to the desired values, with a damping constant of 1 ps.^65^ Newton’s equation of motion was integrated using the Verlet algorithm with a time step of 2 fs. The production simulations were run for 300 ns.^65^ Trajectories were saved every 1 ns. All trajectories were used to determine the formation of atomic Fe and Fe clusters and to calculate the Ar density and the diffusion coefficients for Ar diffusion along the *z*-direction.

### 4.7 Electrochemical Measurements

An electrochemical workstation (CHI760E) was employed to conduct electrochemical measurements in a standard three-electrode system. A graphite rod and a Ag/AgCl (4 M KCl) electrode were utilized as a counter electrode and reference electrode, respectively. An RDE with a glassy carbon disk (5.0 mm diameter), an RRDE electrode with a Pt ring (6.25 mm inner diameter and 7.92 mm outer diameter), and a glassy carbon disk (5.61 mm diameter) were used as working electrodes. The catalyst ink was prepared by dispersing 2.5 mg of catalysts with 490 μL of ethanol and 10 μL of Nafion solution (5 wt %) followed by sonication. A certain volume of ink was pipetted on the disk electrode to yield a uniform film with a catalyst loading of 0.6 mg cm^–2^. The activation process for electrocatalysts was carried out by using cyclic voltammetry (CV) measurements in an O_2_-saturated 0.5 M H_2_SO_4_ solution at a scan rate of 50 mV s^–1^ until the CV profile was stabilized. The ORR polarization curves were recorded at a sweep rate of 5 mV s^–1^ and a rotating rate of 900 rpm. The ring current was measured by the RRDE technique at 1.3 V to calculate the H_2_O_2_ yield and determine the four-electron selectivity. Additionally, experiments at a constant potential were conducted. A commercial Pt/C catalyst (20 wt % Pt; Fuel Cell Store) was utilized as a reference material with a loading of 0.1 mg_Pt_ cm^–2^ in an O_2_-saturated 0.1 M HClO_4_ solution. Catalyst stability was studied by holding at a constant potential of 0.8 V for 60 h during the ORR.

### 4.8 PEMFC Tests

The cathode catalyst (25 mg) was suspended in a mixture of 0.3 mL of Milli-Q water and 0.7 mL of 2-propanol along with 0.6 mL of Nafion ionomer solution (5 wt %, Sigma-Aldrich), and the mixture was sonicated under an ice bath for 90 min. The catalyst inks for the anode and cathode were coated on a Teflon sheet, followed by drying at 80 °C for 5 h. The catalyst layers were then transferred onto the Nafion HP membrane under hot-pressing conditions. The catalyst loading was determined by the weights of Teflon sheets before and after transferring the catalyst layer, and the catalyst loadings at the cathode were 4 mg cm^–2^, while the Pt loading at the anode was 0.2 mg cm^–2^, respectively, under the active area of 5.0 cm^2^. A commercial Pt/C (46.1 wt % Pt, Tanaka Kikinzoku Kogyo K.K., Japan) catalyst was used at the anode. Fuel cell polarization curves were recorded at 100% relative humidity and 80 °C at 2.0 bar H_2_–O_2_ and 2.0 bar H_2_–air backpressures. The PEM fuel cell was conditioned at 0.7 V for 45 min before recording the polarization curves. The flow rates were H_2_/O_2_: 0.3/0.4 nlpm and H_2_/air: 0.3/0.6 nlpm, respectively. The fuel cell was maintained at different voltages to assess the stability.

### 4.9 Ullmann Coupling Reaction

Iodoanisole (1 equiv, 100 μmol, 23.4 mg), the phenol derivative (1.5 equiv), Cs_2_CO_3_ (2 equiv, 200 μmol, 65.1 mg), and Cu catalyst (2.5 mol % Cu) were added into a 10 mL pyrex tube equipped with a stir bar. To that mixture, 1 mL of *N*-methyl-2-pyrrolidone was added. The resulting reaction mixture was dispersed with an ultrasonication bath for 10 min. The tube was closed with a snap cap and submitted to the microwave reactor (Discover SP, CEM). The reaction mixture was heated at 170 °C for 1 h using 200 W under stirring. After completion of the reaction, the solution was diluted with methanol (20 mL), and the catalyst was centrifuged off (15 min, 4800 rpm). The obtained reaction mixture was concentrated in vacuo. The resulting residue was taken up into dichloromethane and submitted to silica column chromatography (hexane/dichloromethane, 8:2) to obtain the isolated product.

### 4.10 Recyclability Tests

Recyclability screening was conducted for the reaction between iodoanisole and phenol following the same procedure of Ullmann couplings. After completion of the reaction completion, 100 μL of a crude reaction mixture was dissolved in 1 mL of methanol. This solution was submitted to GC/MS to quantify the product conversion. The residual crude reaction mixture was dissolved in 30 mL of methanol and submitted to centrifugation (15 min, 4800 rpm). The solvent was decanted. This procedure was repeated 3 times. The obtained black residue was dried under vacuum for 16 h before the next reaction cycle. A total of 4 reaction cycles were carried out.

## Supporting Information Available

Detailed CFD simulation method, experimental information for Ullmann reactions, 1H NMR spectra for all compounds, and extended figures, and tables (PDF)
We further recorded the evolution of Fe atoms with time, suggesting that metal atoms were exchanged between the gas phase and the graphene surface (Video 1) (MP4)
We further recorded the evolution of Fe atoms with time, suggesting that metal atoms were exchanged between the gas phase and the graphene surface (Video 2) (MP4)
We further recorded the evolution of Fe atoms with time, suggesting that metal atoms were exchanged between the gas phase and the graphene surface (Video 3) (MP4)
