# Supplementary material for: Single-Atom Catalysts through Pressure-Controlled Metal Diffusion
Source: J Am Chem Soc. 2024 Jul 11;146(29):19886–95. doi: 10.1021/jacs.4c03066 (PMC11273616; doi:10.1021/jacs.4c03066)
Supplement: Supplementary file 1 — ja4c03066_si_001.pdf [file ja4c03066_si_001.pdf]

Single-atom catalysts through pressure-controlled metal diffusion

Samir H. Al-Hilfi[1,2,#], Xikai Jiang[3,#], Julian Heuer,[2,#] Srinu Akula[4], Kaido Tammeveski[4], Guoqing Hu[5], Juan Yang[1], Hai. I Wang[2,6], Mischa Bonn[2], Katharina Landfester[2], Klaus Muellen[2], Yazhou Zhou[1,2]

1. School of Materials Science and Engineering, Jiangsu University, Zhenjiang, Jiangsu 212013 China

2. Max Planck Institute for Polymer Research, 55128 Mainz, Germany

3. State Key Laboratory of Nonlinear Mechanics, Institute of Mechanics, Chinese Academy of Science, Beijing 100190, China

4. Institute of Chemistry, University of Tartu, Ravila 14a, 50411 Tartu, Estonia

5. Department of Engineering Mechanics, State Key Laboratory of Fluid Power and Mechatronic Systems, Zhejiang University, Hangzhou, Zhejiang 310027, China

6. Nanophotonics, Debye Institute for Nanomaterials Science, Utrecht University, Princetonplein 1, 3584 CC Utrecht, The Netherlands

[#]Samir H. Al-Hilfi, Xikai Jiang, and Julian Heuer contributed equally.

E-mail: bonn@mpip-mainz.mpg.de, muellen@mpip-mainz.mpg.de, and yazhou@mpip-mainz.mpg.de

This file includes:

Figures. S1 to S31

Tables S1 to S5

CFD simulation method

Boundary layer thickness calculations

Organic synthesis

NMR

GC

References

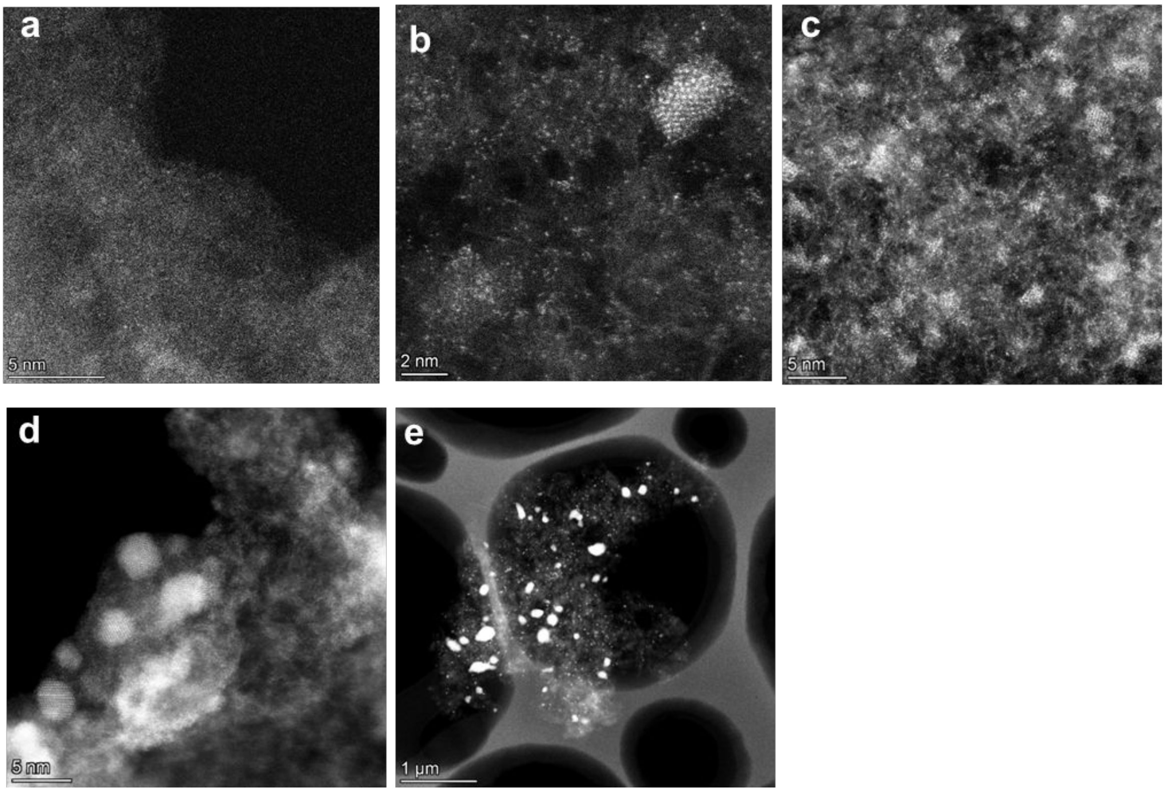

Supplementary Fig. 1. HAADF-STEM images of Fe-NC prepared under ambient pressure with the mass ratio of NaFe-EDTA/NC, (a) 0.08, (b) 0.1, (c) 0.2, (d) 0.3, and (e) 0.36.

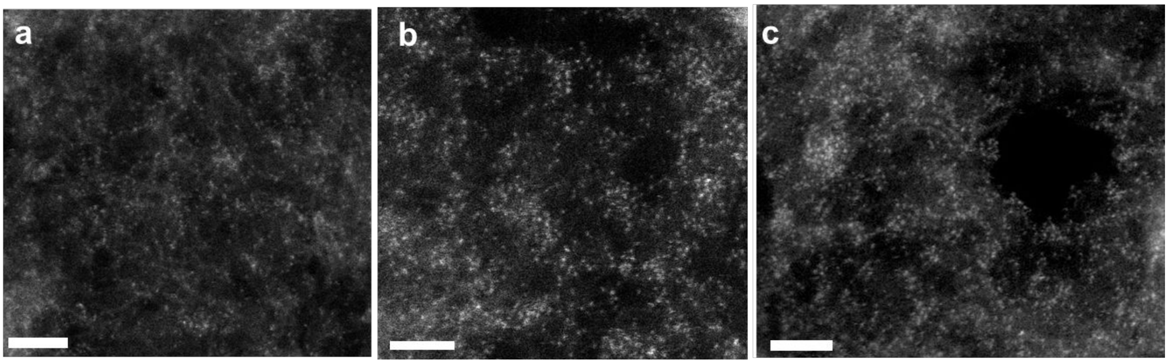

Supplementary Fig. 2. Aberration-corrected HAADF-STEM images of (a) $Fe_{0.2}$-$NC^{1.4}$, (b) $Fe_{0.2}$-$NC^{0.52}$, and (c) $Fe_{0.2}$-$NC^{0.14}$. Scale bar: 2 nm.

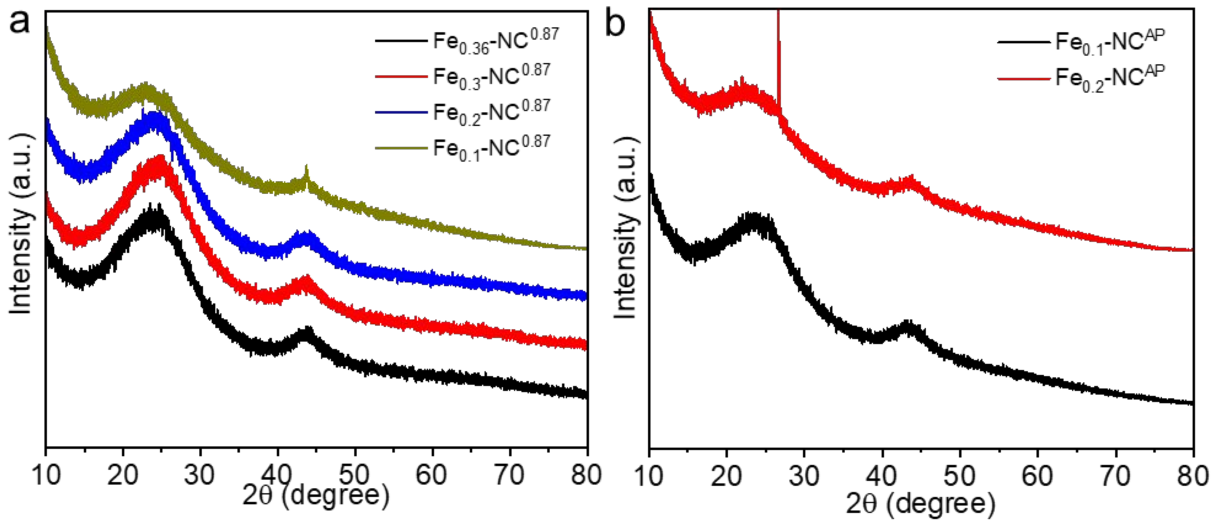

Supplementary Fig.3. XRD analysis of (a) Fe-NC prepared under 0.87 mbar with various mass ratios of NaFe-EDTA/NC and (b) Fe-NC prepared under ambient pressure with mass ratios of NaFe-EDTA/NC of 0.1 and 0.2.

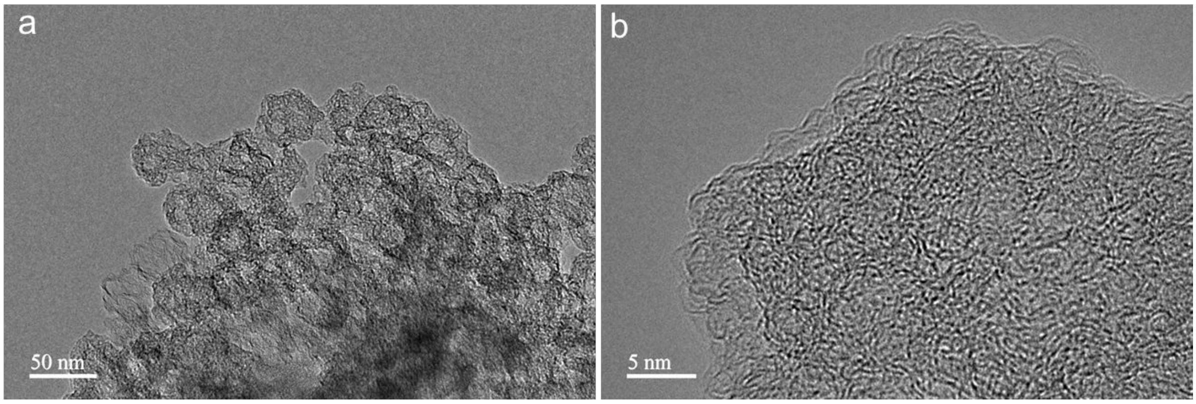

Supplementary Fig.4. (a) TEM and (b) HRTEM images of $Fe_{0.3}$-$NC^{0.87}$.

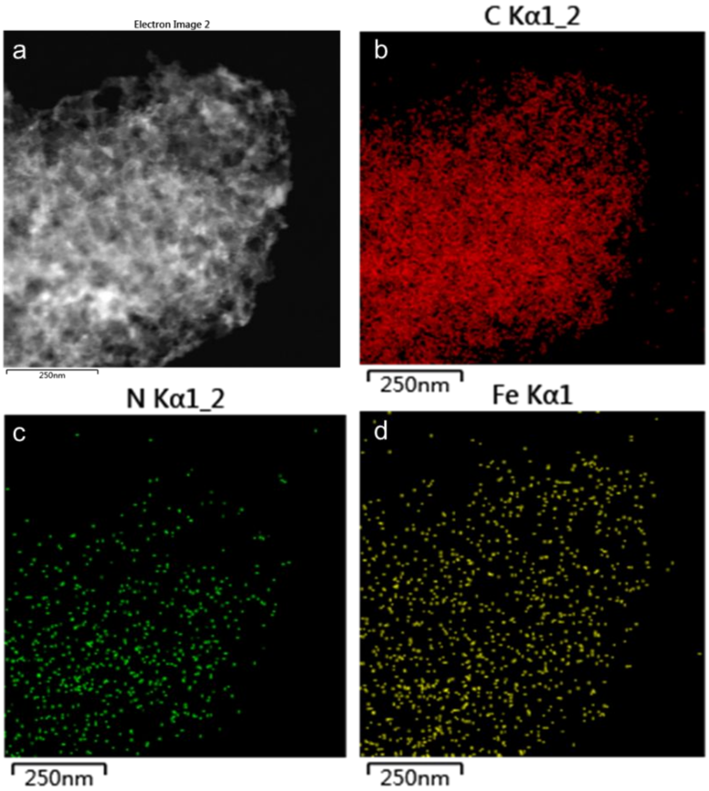

Supplementary Fig. 5. (a) HAADF-STEM and corresponding elemental images showing the distribution of (b) C, (c) N, and (d) Fe elements for $Fe_{0.3}$-$NC^{0.87}$.

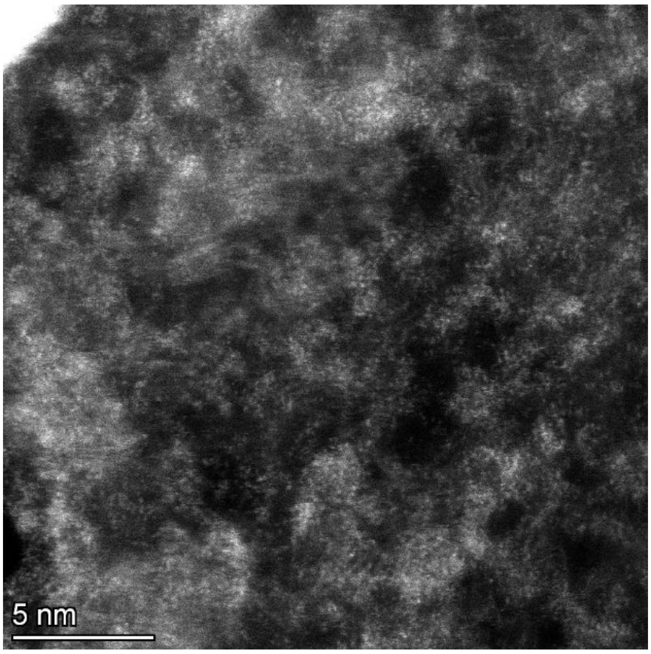

Supplementary Fig. 6 Aberration-corrected HAADF-STEM image of $Fe_{0.36}$-$NC^{0.87}$.

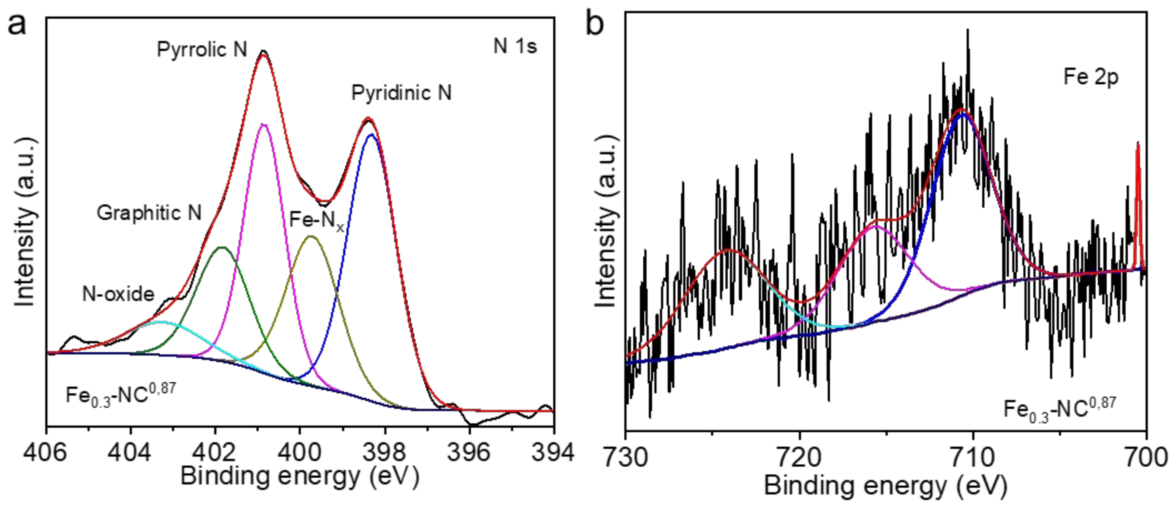

Supplementary Fig. 7. XPS analysis for $Fe_{0.3}$-$NC^{0.87}$ (a) High-resolution N 1s XPS spectrum and High-resolution Fe_{2p} XPS spectrum.

Supplementary Table 1. Fe loadings in $Fe_{0.08}$-NC^{AP} $Fe_{0.2}$-$NC^{0.87}$, and $Fe_{0.3}$-$NC^{0.87}$, according to ICP analyse.

**Catalyst**	**Fe(wt%)**	**Zn(wt%)**
NC	/	< 0.1
$Fe_{0.08}$-NC^{AP}	1.4	< 0.1
$Fe_{0.2}$-$NC^{0.87}$	3.2	/
$Fe_{0.3}$-$NC^{0.87}$	4.5	/

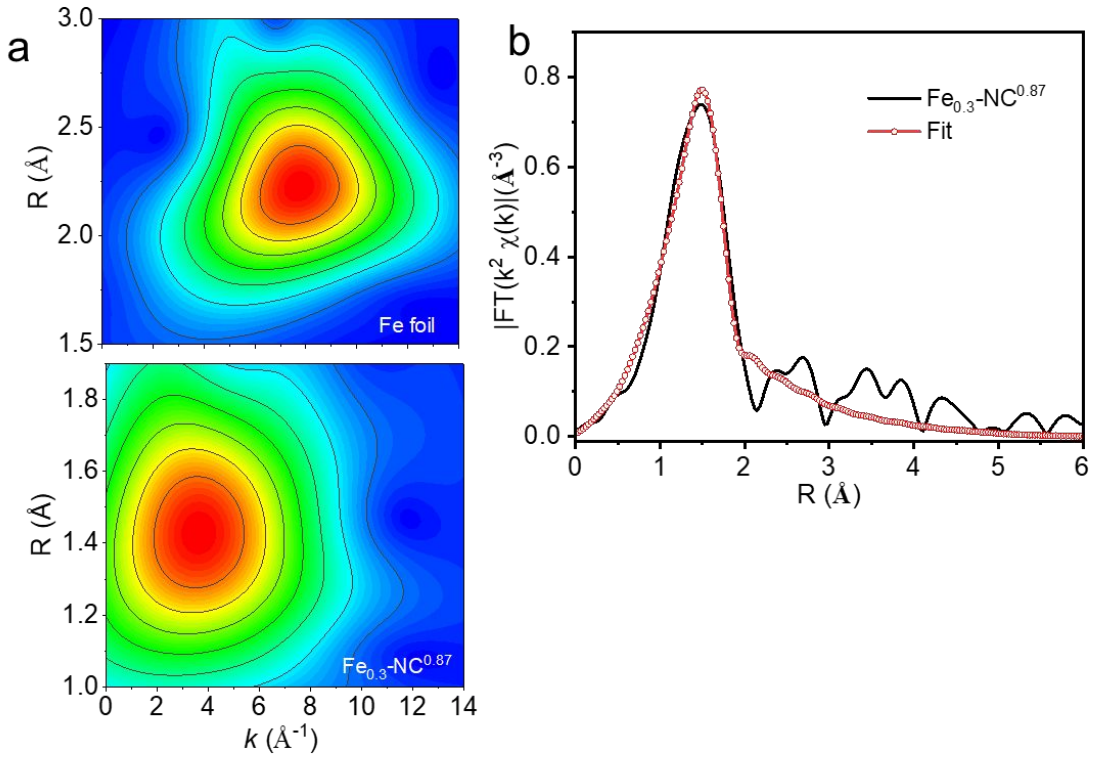

Supplementary Fig. 8. (a) The wavelet transform (WT) of Fe K-edge EXAFS spectra of $Fe_{0.3}$-$NC^{0.87}$ and Fe foil. (b) The Fourier transform (FT) of the EXAFS spectra of $Fe_{0.3}$-$NC^{0.87}$ and its fit of $Fe_{0.3}$-$NC^{0.87}$.

Supplementary Table 2. Structural parameters of the $Fe_{0.3}$-$NC^{0.87}$ and Fe foil reference extracted from the EXAFS fitting.

**Sample**	**Path**	***N***	***R* (Å)**	**σ^2 ($\times 10^{-3}$ Å^2)**	**ΔE_0 (eV)**	***R*, %**
Fe foil[a]	Fe-Fe	**8**	2.47±0.01	5±1	6±2	0.53
	Fe-Fe	**6**	2.85±0.01	6±2		
Fe-EDTA-LP[b]	Fe-N/O	6.1±1.1	2.00±0.02	11±3	-5±2	1.41

[a]: *k* range: 3-13.6 (Å^{-1}); *R* range: 1-3 Å; [b]: *k* range: 3-12 (Å^{-1}); *R* range: 1.0-2 Å; $S_0^2 = 0.68$ and determined from Fe foil. The bolded numbers represent fixed coordination numbers.

Supplementary Table 3. Porosity properties of the NC and $Fe_{0.3}$-$NC^{0.87}$.

**Sample**	**S_{micro} m^2 g^{-1}**	**$S_{external}$ m^2 g^{-1}**	**Micropore volume cm^3 g^{-1}**	**Total specific surface area (m^2 g^{-1})**
NC	463	1006	0.13	1490
$Fe_{0.3}$-$NC^{0.87}$	433	735	0.10	1168

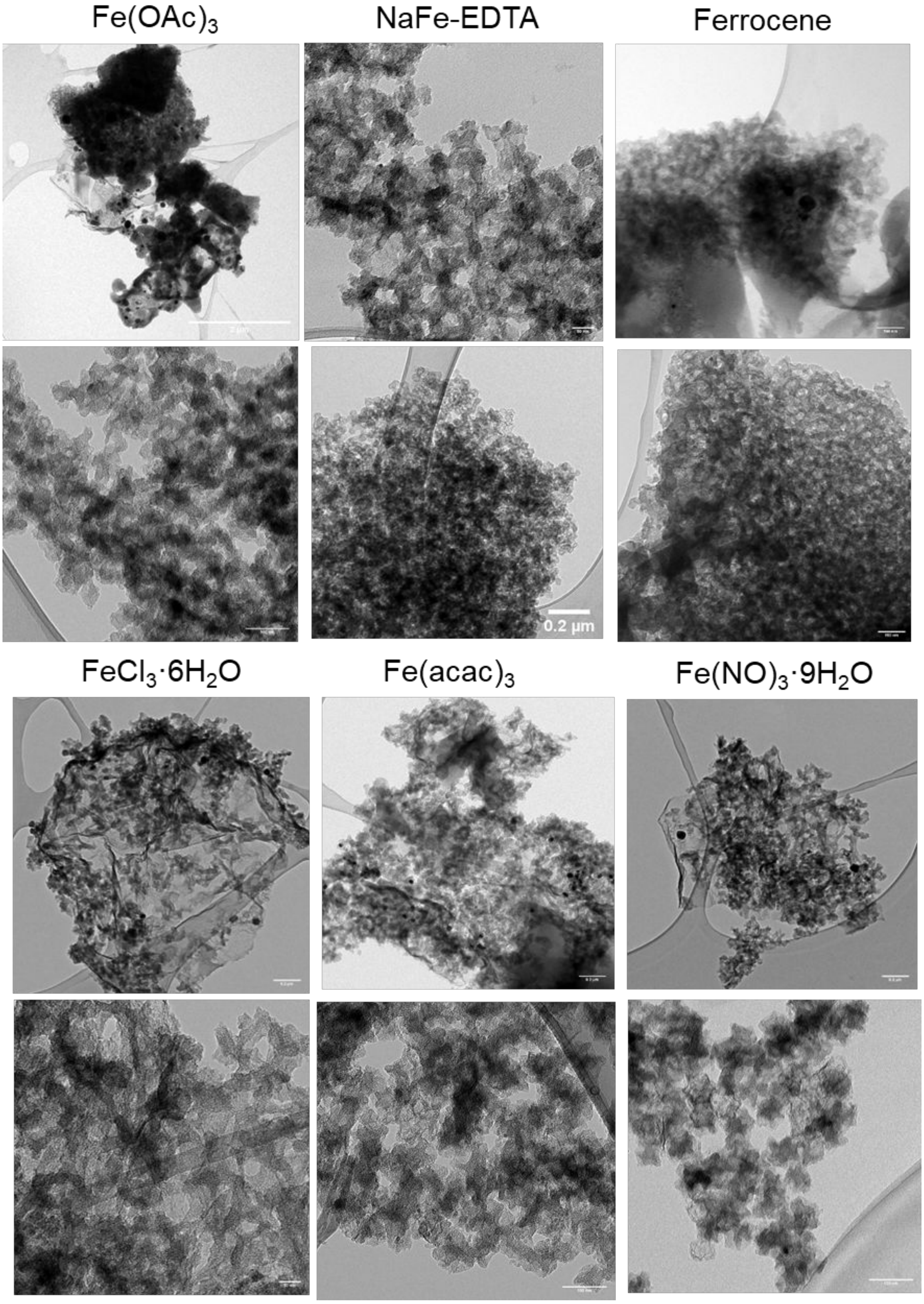

Supplementary Fig. 9. TEM images of Fe-NC materials prepared from the pyrolysis of a mixture of NC and various Fe precursors under ambient pressure (Up) and 0.87 mbar (Bottom), respectively. The mass ratios between NC and Fe precursors are 0.2 for $FeCl_3 \cdot 6H_2O$, 0.26 for $Fe(acac)_3$, 0.2 for $Fe(NO_3)_3. \cdot 9H_2O$, 0.26 for $Fe(OAc)_3$, 0.3 for NaFe-EDTA, and 0.22 for Ferrocene, respectively.

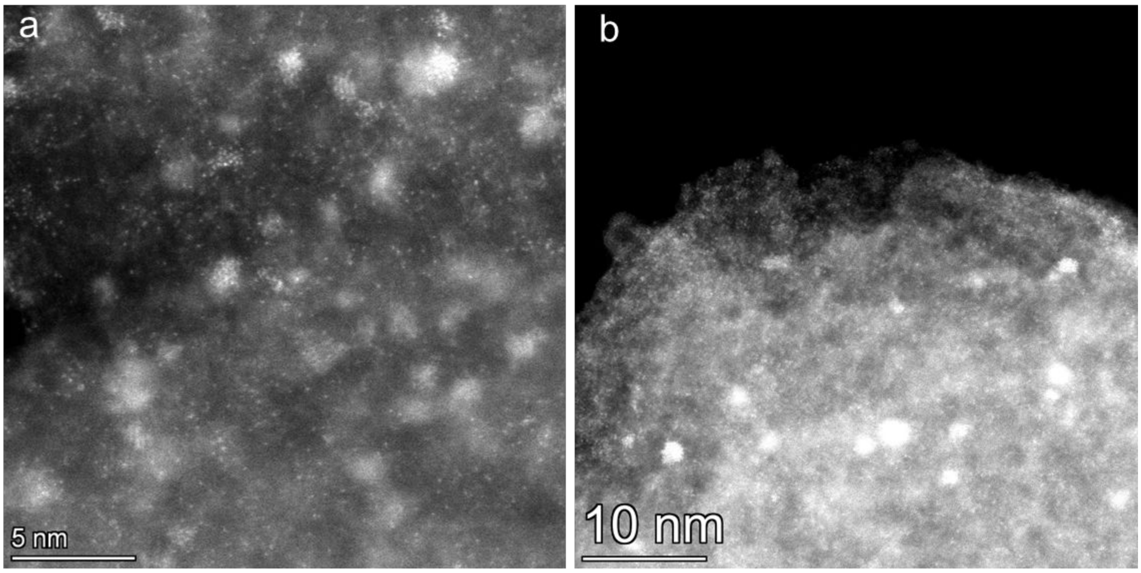

Supplementary Fig. 10. Aberration-corrected HAADF-STEM images of (a) Pd and (b) Ru samples prepared by pyrolysis of Pd(acac)$_2$/NC and Ru(acac)$_3$ under ambient pressure. The mass ratio between metal precursors and NC is 0.24.

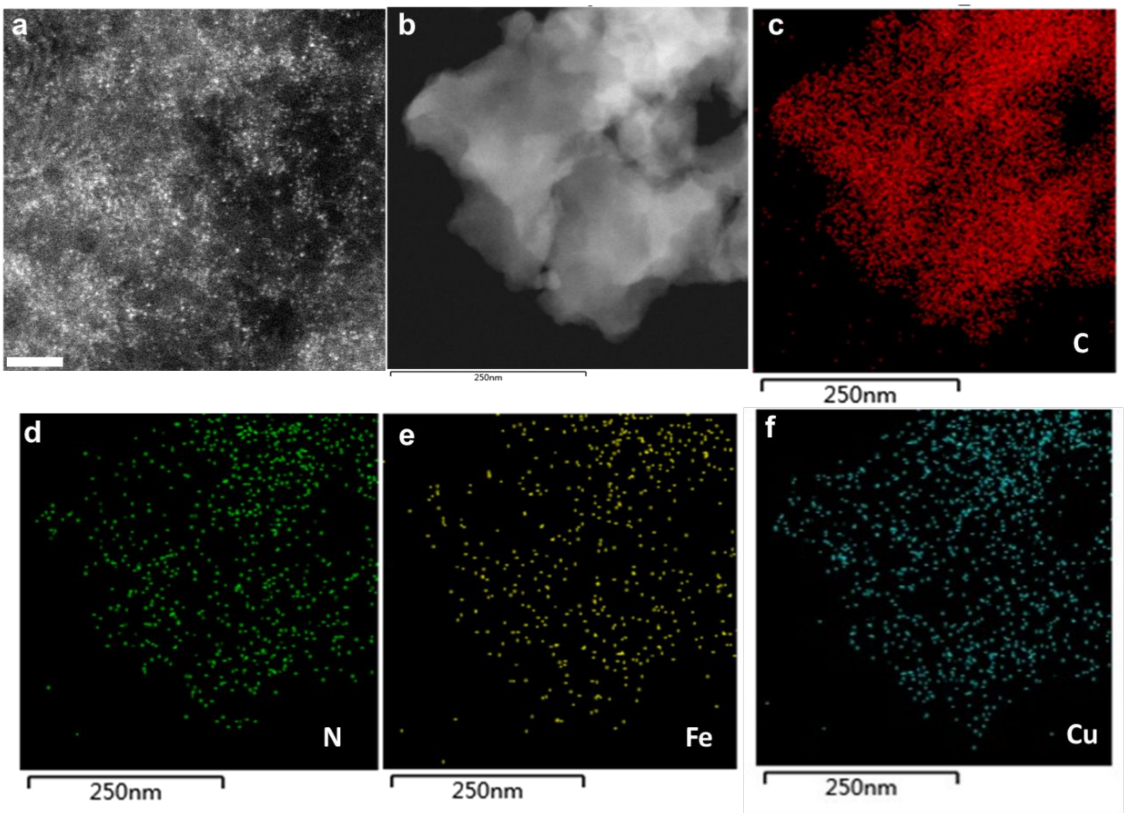

Supplementary Fig. 11. Characterization of Fe,Cu-NC catalyst. (a) Aberration-corrected HAADF-STEM image, (b) STEM image with elemental maps of (c) C, (d) N, (e) Fe, and (f) Cu. Scale bar in a: 2 nm.

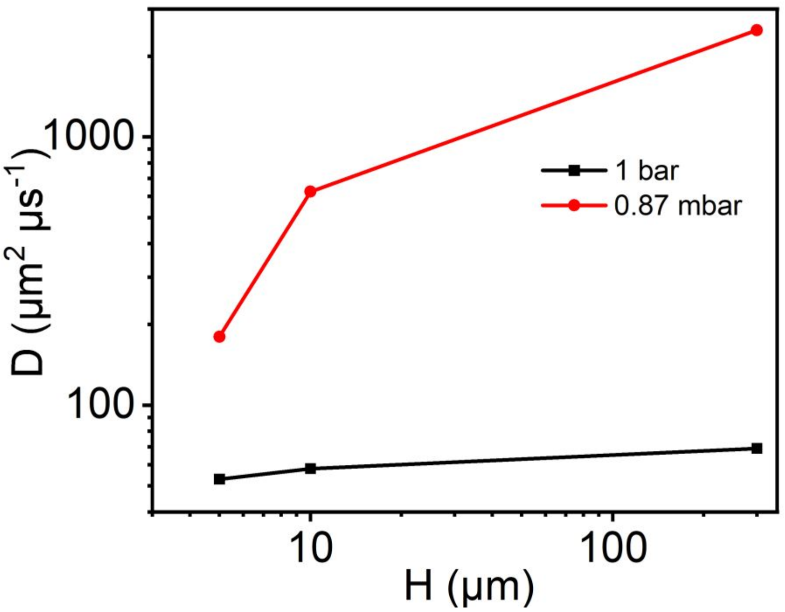

Supplementary Fig. 12. Correlations of the Fe atom diffusion coefficient with the distance of neighboring graphene sheets at 900 ºC and pressure of 1 bar and 0.87 mbar.

CFD simulations. CFD simulations were performed using COMSOL Multiphysics. We used the real reactor geometry, dimensions, and working conditions in 2D space (Supplementary Fig. 14). The geometry has an inlet and outlet point and the boundary conditions were set based on the experimental pressures. The material is quartz tube and Ar gas carrier and their properties are provided by the software library. The temperature of the central tube is 900 °C and two ends are subjected to external natural convection. The geometry meshed to 46134 elements, 42882 triangles, 3252 quads, 1662 edges, and 16 vortexes. The fluid flow that was assumed weakly compressible, was simulated based on the solutions of the Navier–Stokes equation. To probe the change in fluid properties related to the temperature, the fluid flow and heat transfer interfaces were coupled to a non-isothermal flow multiphysics interface. The model was run under the stationary state. The resulting fluid properties (i.e., velocity, density, and dynamic viscosity) were used to calculate the Reynolds number. Thus, the boundary layer thickness was obtained (Supplementary Table 4).

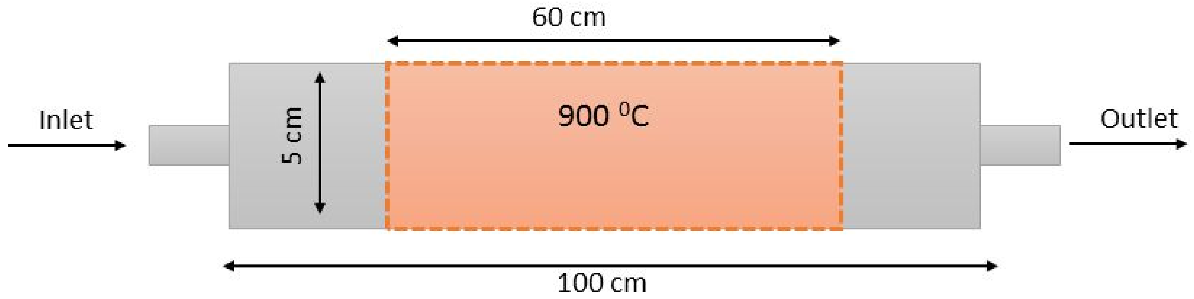

Supplementary Figure 13. 2-D schematic diagram of the working conditions of the pyrolysis reactor used in this study. The reactor is 100 cm in length and 5 cm in diameter with a 60 cm length heating zone at 900 °C. The reactor has an inlet point, where the gas is introduced to the system, and an outlet point connected to a mechanical pump to regulate the reactor pressure.

Boundary layer thickness calculations: This calculation is based on equation S1[1,2](R. B. Bird, 2002) (M. K. Z. Daniel M. Dobkin 2003)

$$Re(L) = \frac{\rho v L}{\mu} \quad \textbf{\textit{(S1)}}$$

where ρ is the gas density, v is the mean velocity of the flow, and L denotes the characteristic length, set to the vessel radius, □. μ is the dynamic viscosity. The ideal gas model and the principle of conservation of mass have been used to convert the volumetric flow rate at which gases are introduced into the chamber (F_s), to the expected flow rate within the chamber (F_r). v is then calculated by dividing the cross-sectional area of the reaction chamber.

$$v = \frac{F_S P_S T_r}{T_s P_r \pi r^2} \quad \textbf{(S2)}$$

For these calculations, it is assumed that F_s is measured at standard temperature and pressure. The gas density is calculated using the ideal gas law as shown in the equation S3:

$$\rho = \frac{P_r}{K_B T_r} \quad \textit{(S3)}$$

where k_B is Boltzmann's constant. The dynamic viscosity is related to the temperature and molecular properties of a given species of gas according to equation S4[3] (X.-T. Y. Yongdong Xu 2010).

$$\mu = \frac{2}{3\pi^2 d^2}\sqrt{\pi m K_B T_r} \quad \textit{(S4)}$$

where d is the molecular diameter. Combining equations S1-4 shows that in this model, the Reynolds number could be calculated for our reactor geometry (Figure 1) as follows:

$$Re(r) = \frac{3F_S P_S d^2}{2T_S r}\sqrt{\frac{\pi m}{K_B^3 T_r}} \quad \textit{(S5)}$$

The average thickness of the boundary layer over a substrate of length Ls, δ, is related to the Reynolds number according to the equation S6:

$$\bar{\delta} = \frac{10}{3}\frac{L}{\sqrt{Re_r}} \quad \textit{(S6)}$$

The Reynolds numbers and the average boundary layer thickness are listed in Table S4.

Supplementary Table 4. Calculated gas flow parameters for the reactor geometry in this study under different pressures.

**Pressure (mbar)**	**Velocity (m s^{-1})**	**Density (g m^{-3})**	**Re**	**Boundary layer (cm)**
0.14	17.21	0.12	2.47	2.1
0.52	19.85	0.21	4.94	1.5
0.87	20.98	0.34	8.23	1.1
1.4	24.58	0.57	16.47	0.8
1010	0.002	0.41	0.82	3.7

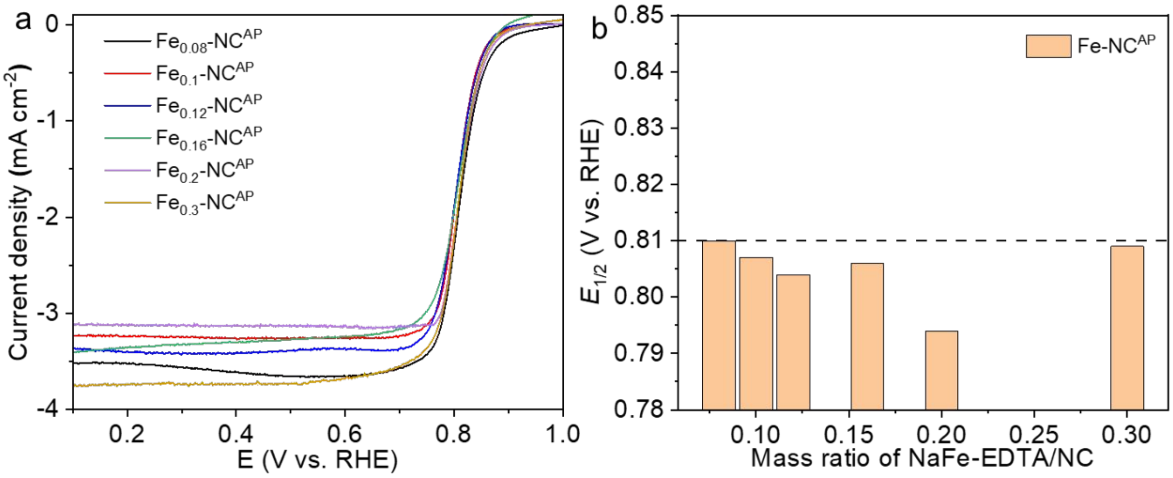

Supplementary Fig. 14. (a) ORR polarization curves of Fe-NC catalysts prepared using different amounts of NaFe-EDTA at ambient pressure, (b) the correlation of $E_{1/2}$ value with the mass ratio of NaFe-EDTA/NC.

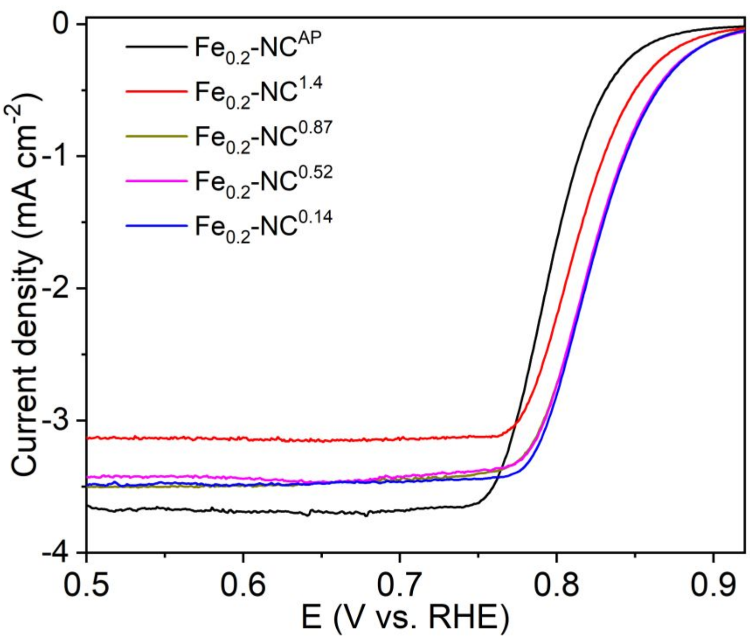

Supplementary Fig. 15. ORR polarization curves of (a) $Fe_{0.2}$-NC^{y} catalysts prepared under various pressures. This measurement was carried out in O_2-saturated 0.5 M H_2SO_4 at 5 mV s^{-1} with a rotation speed of 900 rpm.

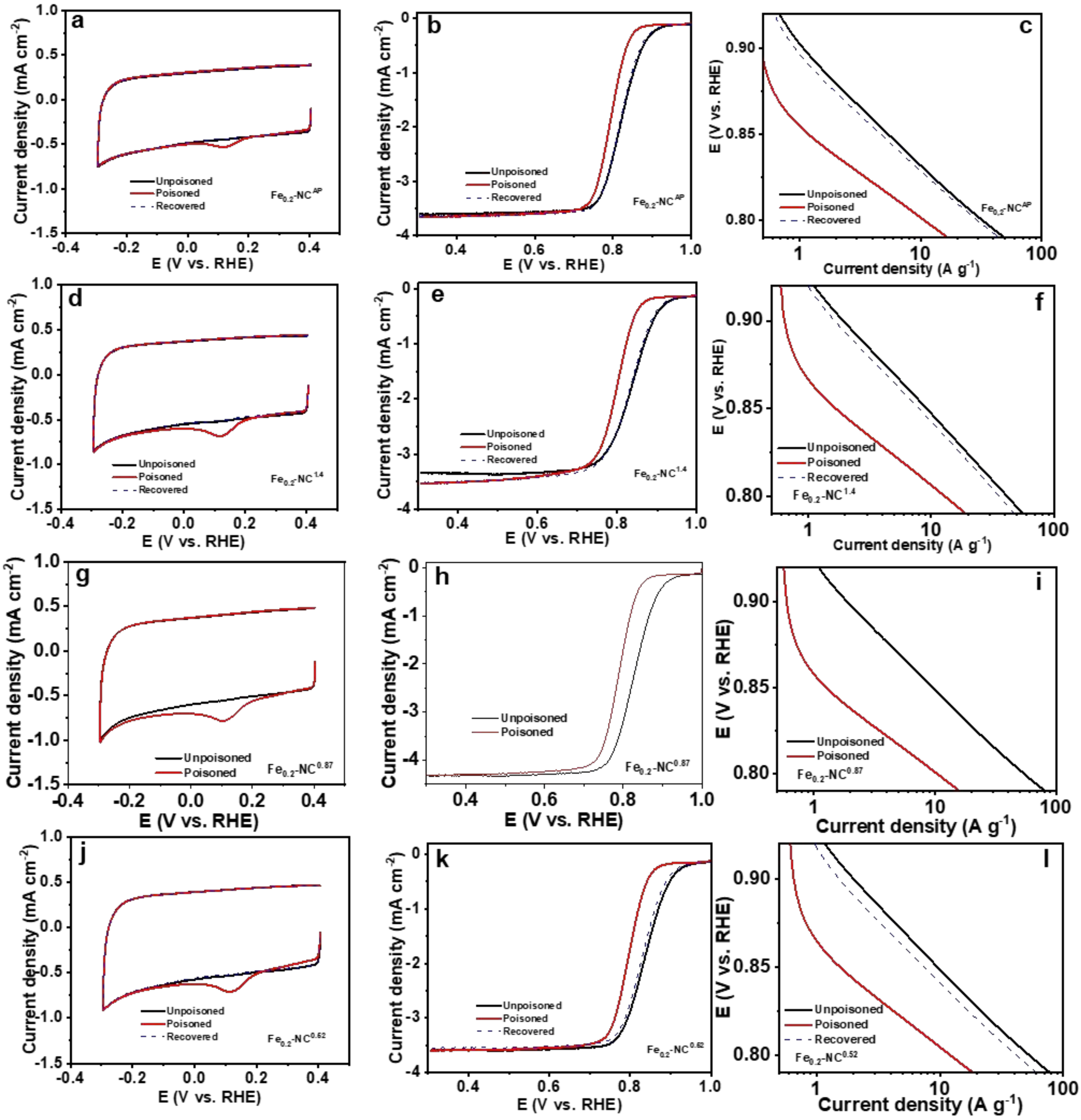

Supplementary Fig. 16. Determination of SD_{mass} of $Fe_{0.2}$-NC catalysts prepared at different pressures through reversible nitrite poisoning in 0.5 M acetate buffer at pH 5.2 at room temperature. Left column, CV curves before, during, and after nitrite adsorption in the nitrite reductive stripping region; middle column, LSV curves before, during and after nitrite adsorption, the RDE data were collected in O_2-saturated acetate buffer with a scan rate of 10 $mV \cdot s^{-1}$ and a rotation rate of 900 rpm. Right column, the corresponding kinetic current density plots. The catalyst loading was 270 μg cm^{-2}.

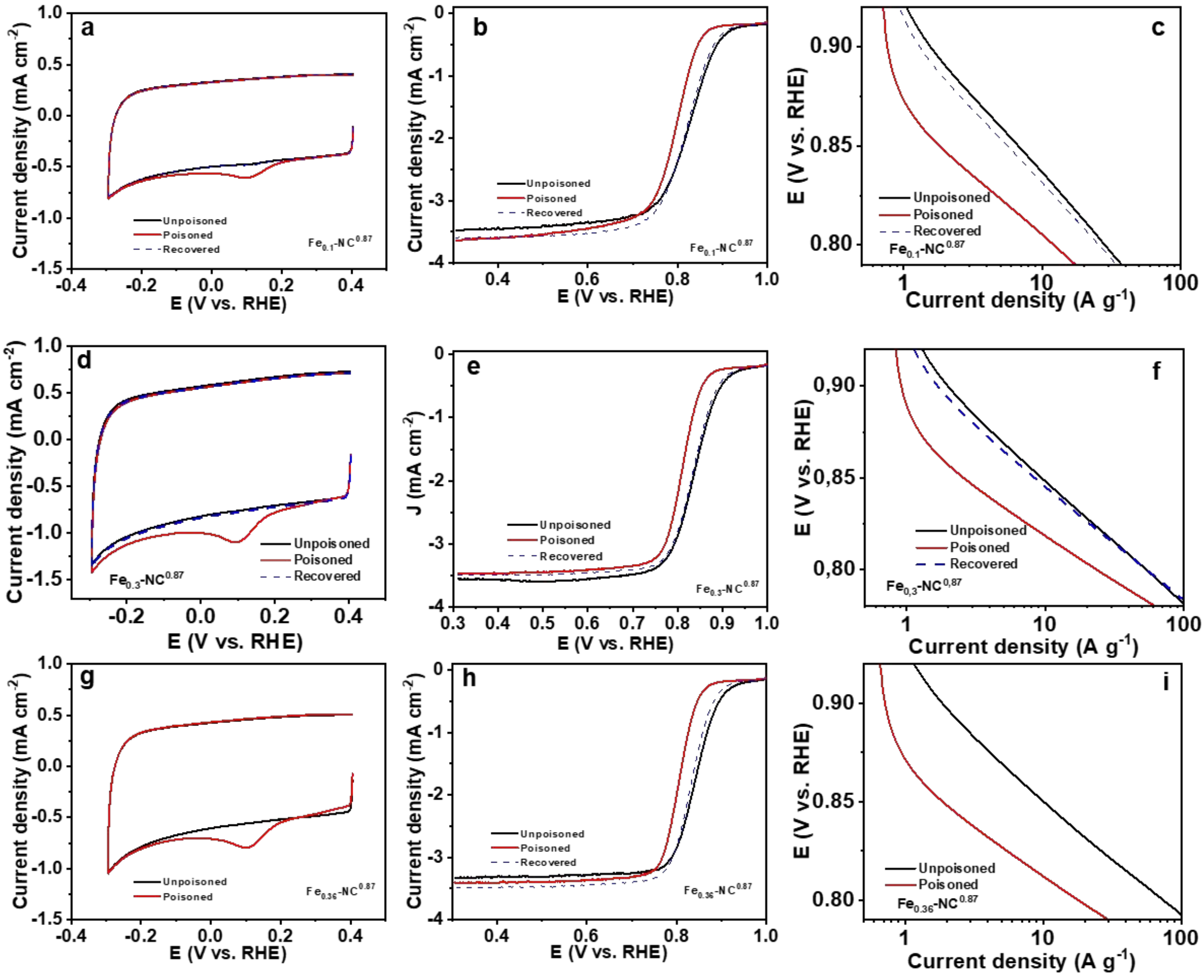

Supplementary Fig. 17. Determination of SDmass of Fe-NC$^{0.87}$ catalysts prepared using different amounts of NaFe-EDTA through reversible nitrite poisoning in 0.5 M acetate buffer at pH 5.2 at room temperature. Left column, CV curves before, during, and after nitrite adsorption in the nitrite reductive stripping region; middle column, LSV curves before, during, and after nitrite adsorption, the RDE data were collected in O_2-saturated acetate buffer with a scan rate of 10 mV·s^{-1} and a rotation rate of 900 rpm. Right column, the corresponding kinetic current density plots. The catalyst loading was 270 μg cm^{-2}.

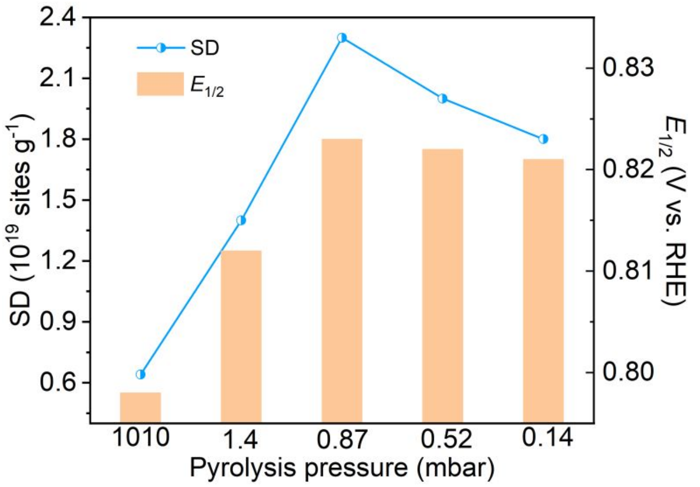

Supplementary Fig. 18. Correlation of ORR activity and site density on $Fe_{0.2}$-NC^{y} catalysts.

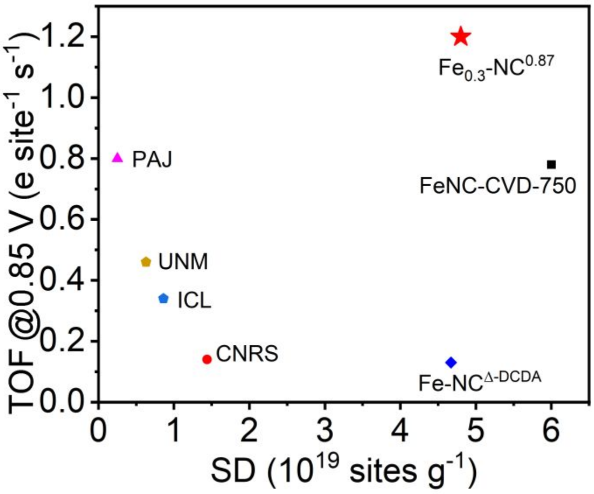

Supplementary Fig. 19. Comparison of site density and TOF of $Fe_{0.3}$-$NC^{0.87}$ at 0.85 V with reported values. PAJ, UNM, ICL, CNRS[4],FeNC-CVD-750[5], and Fe-NC$^{\Delta\text{-DCDA}}$ [6]

Supplementary Table 5. Summary of catalyst site density and TOF by the nitrite stripping experiment

Catalyst	Method	$\Delta j_{0.85}$	$\Delta j_{0.9}$	$TOF_{0.85\,V}$ ($e^{-}\cdot site^{-1}\cdot s^{-1}$)	$TOF_{0.9\,V}$ ($e^{-}\cdot site^{-1}\cdot s^{-1}$)	SD (10^{19} sites g^{-1})
$Fe_{0.2}$-NC^{AP}	NO_2^-	4.2	0.5	4.1	0.5	0.64
$Fe_{0.2}$-$NC^{1.4}$	NO_2^-	7.6	1.3	3.4	0.6	1.4
$Fe_{0.2}$-$NC^{0.87}$	NO_2^-	9	1.3	2.4	0.4	2.3
$Fe_{0.2}$-$NC^{0.52}$	NO_2^-	8	1.3	2.5	0.4	2
$Fe_{0.2}$-$NC^{0.14}$	NO_2^-	8	1.3	2.8	0.5	1.8
$Fe_{0.1}$-$NC^{0.87}$	NO_2^-	5	0.8	3.1	0.5	1
$Fe_{0.3}$-$NC^{0.87}$	NO_2^-	9	1.3	1.2	0.2	4.8
$Fe_{0.36}$-$NC^{0.87}$	NO_2^-	8	1.2	2.3	0.3	2.2

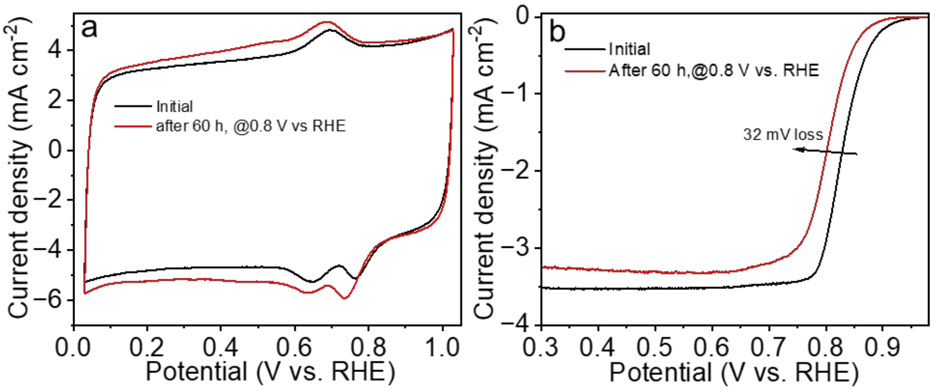

Supplementary Fig. 20. Cyclic voltammetry curves and ORR polarization curves before and after constant potential test at 0.8 V for 60 h.

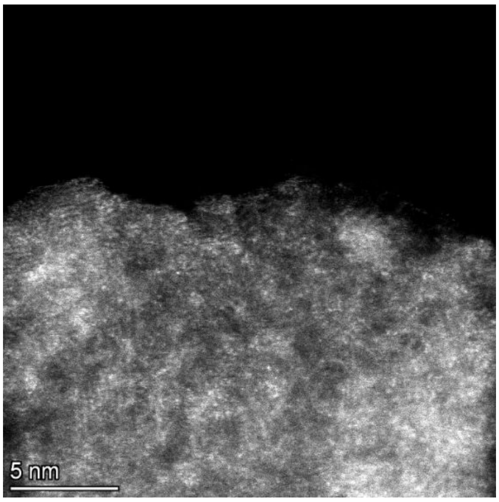

Supplementary Fig. 21. AC-HAADF-STEM image of $Fe_{0.3}$-$NC^{0.87}$ after stability test.

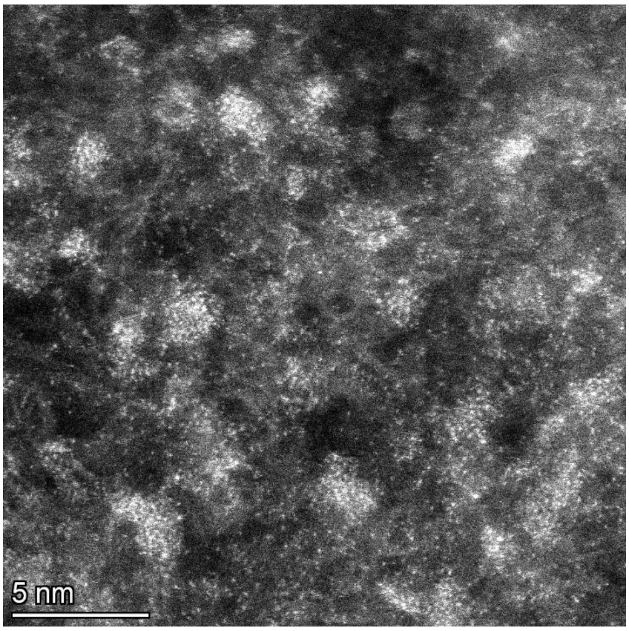

Supplementary Fig. 22. AC-HAADF-STEM image of Cu-NC^{AP}.

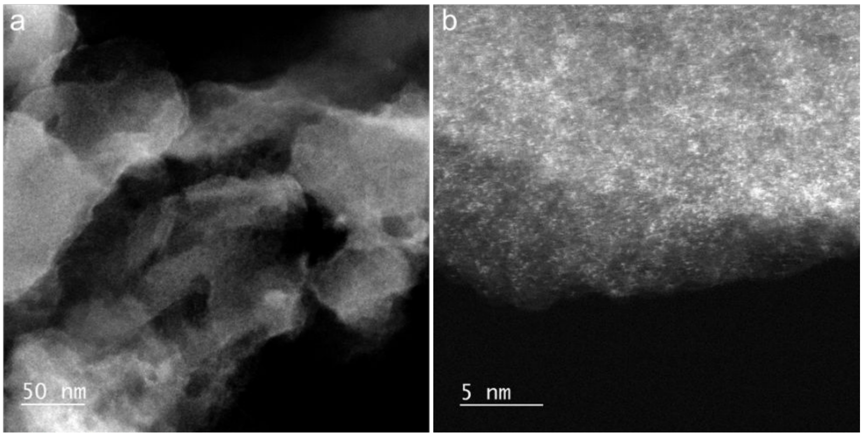

Supplementary Fig. 23. (a) STEM and (b) AC-HAADF-STEM image of Cu-NC$^{0.87}$ after 4-cycle Ullmann reaction.

General Experimental information. ^{1}H- and ^{13}C-NMR spectra were recorded at room temperature on a Bruker Avance III HD 400 spectrometer (^{1}H: 400.13 MHz; ^{13}C: S 4 100.62 MHz), in deuterated solvents (> 99.5 Deuteration) purchased from Sigma-Aldrich, stored at 4 ˚C ($CDCl_3$, DMSO-d6). Chemical shifts (δ) for ^{1}H and ^{13}C NMR spectra were referenced against TMS (tetramethylsilane) and are given in parts per million (ppm). First-order multiplicities in ^{1}H NMR signals were reported using the following abbreviations: s = singlet, d = doublet, t = triplet, q = quartet, p = quintet, h =sextet; m = multiplet, br = broad signal. Data processing of NMR spectra was done with MestReNova 15.0.0

Flash column chromatography was performed on Machery-Nagel 60 M silica gel (40-63 microns) under pressure gradients using the Biotage® Isolera One Purification system, within Biotage® KP-Sil cartridges of differing sizes. Product elution was analyzed by the internal UV detector (200 -800 nm). Thin layer chromatography was conducted using Machery-Nagel Alugram® SIL G/UV_{254} pre-coated aluminum sheets and was visualized by UV light (254 nm or 366 nm), Iodine chamber or stained with basic potassium permanganate solution (1.5 g $KMnO_4$, 10 g K_2CO_3, 1.25 mL 10% NaOH in 200 mL of distilled water).

Gas chromatographic analysis was conducted via a Shimadzu GC-2010 plus GC- system equipped with a 7HG-G010-11 Phenomenex column (0.25 mm x 30 m (diameter: length), film thickness: 0.25 μm) and analyzed using QP2010 ultra mass spectrometer. Using splitless injection, injection temperature 280 ˚C, column oven 100 ˚C. Temperature gradient: 100 ˚C to 280 ˚C with a rate of 10 ˚C/min hold for 5 min Total flow at 19.6 mL/min, 43.6 kPa Pressure, 0.70 mL/min column flow. Preparation of GC/MS samples was performed by the addition of

100 μL of crude solution mixture into 1 mL methanol and then transferred into a 2 mL GC vial. GC-MS spectra were analyzed with GCMSolution Postrun Analysis (Version: 4.45 SP1).

Synthesis of compound 1

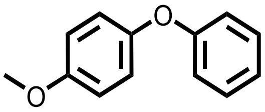

Iodoanisole (1 Eq., 100 µmol, 23.4 mg), phenol (1.5 Eq., 150 µmol, 14.12 mg), Cs_2CO_3 (2 Eq., 200 µmol, 65.1 mg) and Cu catalyst (2.5 mol% Cu) were used for the synthesis of this compound. The general procedure for Ullmann couplings was followed.

^{1}H-NMR (400 MHz, $CDCl_3$, δ): 6.86 (m, 9H, Ar H), 3.72 (s, 3H, CH_3);

^{13}C-NMR (101 MHz, $CDCl_3$, δ): 129.6 (2C, Ar C), 122.5 (1C, Ar C), 120.8 (2C, Ar C), 117.5 (2C, Ar C), 114.9 (2C, Ar C), 55.8 (1C, CH_3);

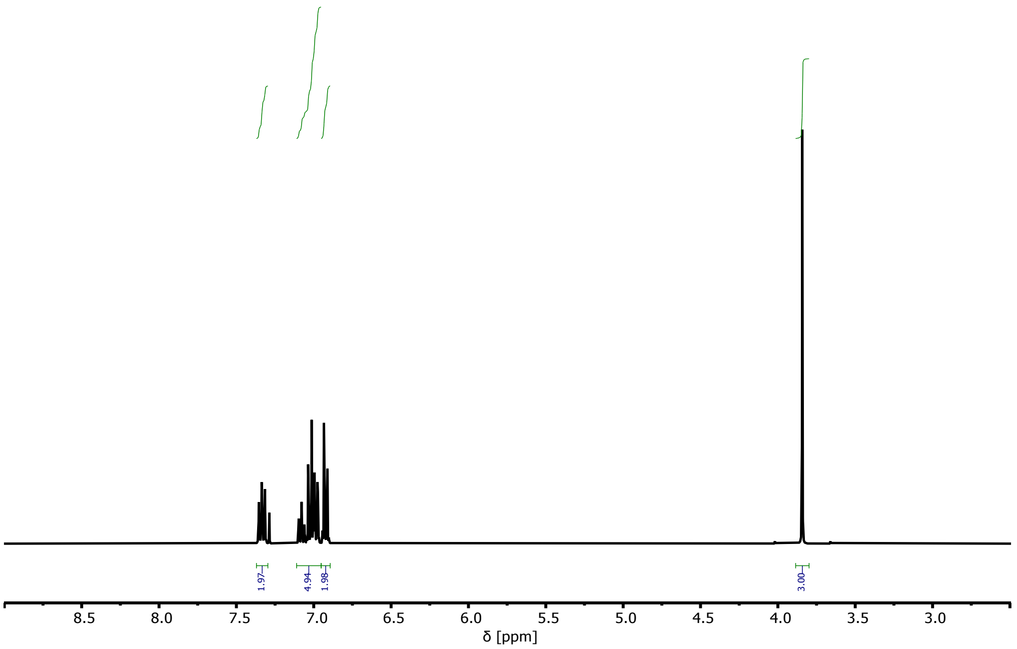

Supplementary Fig.24: ^{1}H-NMR of compound 1.

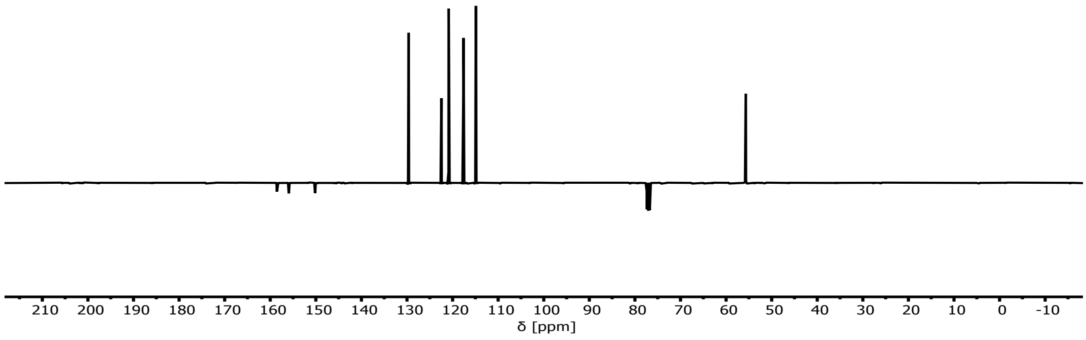

Supplementary Fig. 25: ^{13}C-NMR of compound 1.

Synthesis of compound 2

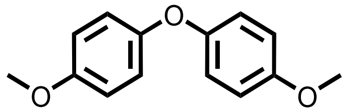

Iodoanisole (1 Eq., 100 µmol, 23.4 mg), 4-methoxyphenol (1.5 Eq., 150 µmol, 18.62 mg), Cs_2CO_3 (2 Eq., 200 µmol, 65.1 mg) and Cu catalyst (2.5 mol% Cu) were used for the synthesis of this compound. The general procedure for Ullmann couplings was followed.

^{1}H-NMR (400 MHz, $CDCl_3$, δ): 6.82 (m, 8H, Ar H), 3.72 (s, 6H, CH_3);

^{13}C-NMR (101 MHz, $CDCl_3$, δ): 119.5 (4C, Ar C), 114.6 (4C, Ar C), 55.6 (2C, CH_3);

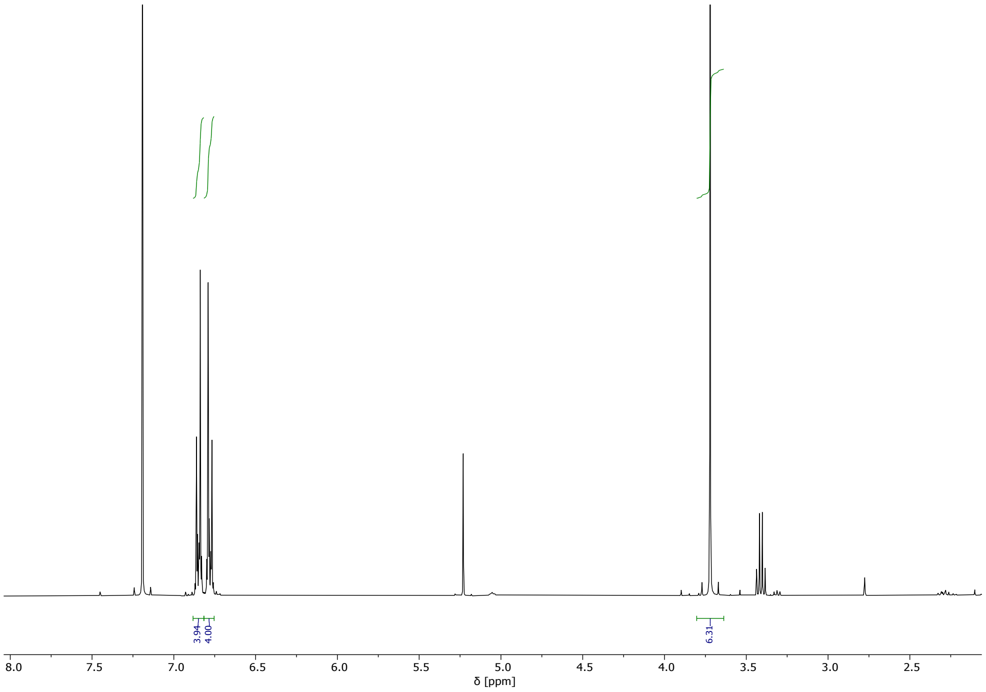

Supplementary Fig. 26: ^{1}H-NMR of compound 2.

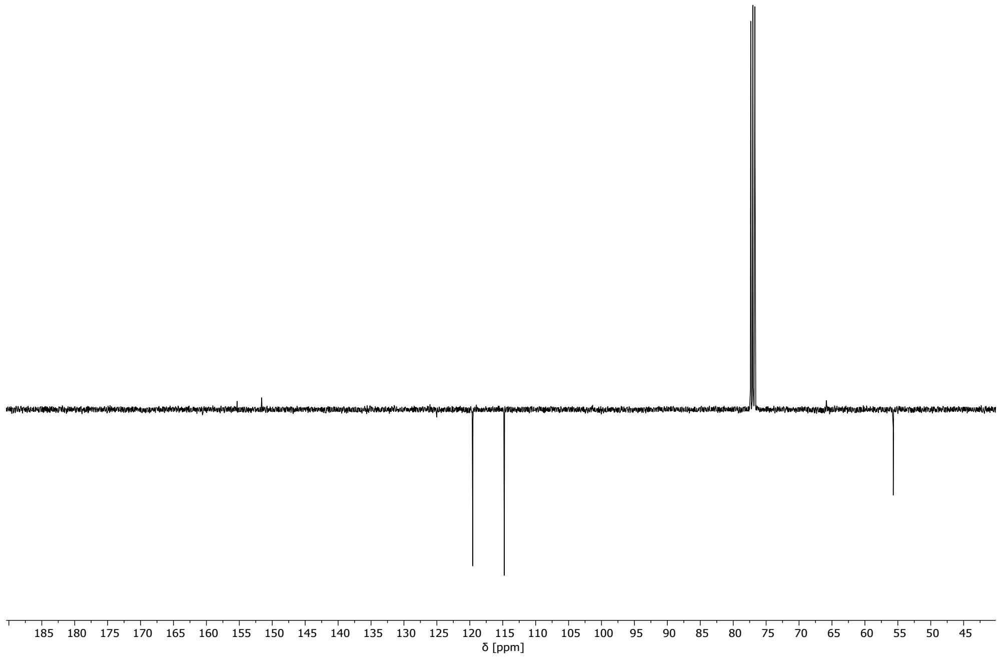

Supplementary Fig. 27: ^{13}C-NMR of compound 2.

Synthesis of compound 3

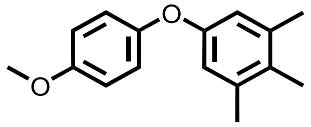

Iodoanisole (1 Eq., 100 µmol, 23.4 mg), 2,3,4-trimethylphenol (1.5 Eq., 150 µmol, 20.43 mg), Cs_2CO_3 (2 Eq., 200 µmol, 65.1 mg) and Cu catalyst (2.5 mol% Cu) were used for the synthesis of this compound. The general procedure for Ullmann couplings was followed.

^{1}H-NMR (400 MHz, $CDCl_3$, δ): 6.83 (m, 4H, Ar H), 6.54 (s, 2H, Ar H) 3.72 (s, 3H, CH_3), 2.16 (s, 6H, CH_3), 2.04 (s, 3H, CH_3);

^{13}C-NMR (101 MHz, $CDCl_3$, δ): 120.5 (4C, Ar C), 116.9 (4C, Ar C), 114.7 (2C, Ar C) 56.0 (1C, CH_3), 41.6 (1C, CH_3), 33.9 (2C, CH_3);

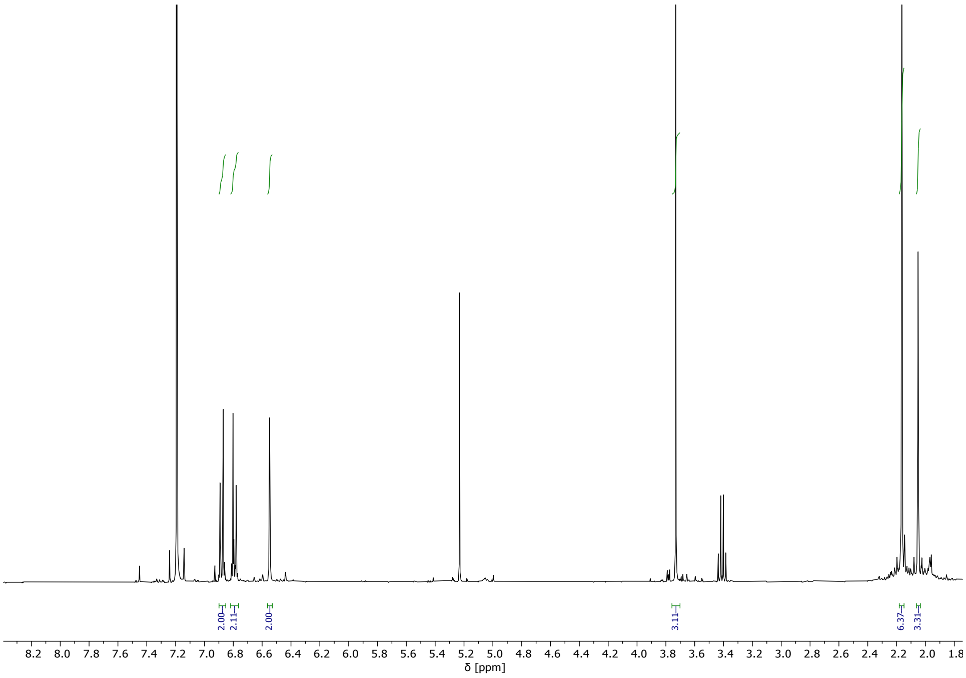

Supplementary Fig. 28. ^{1}H-NMR of compound 3.

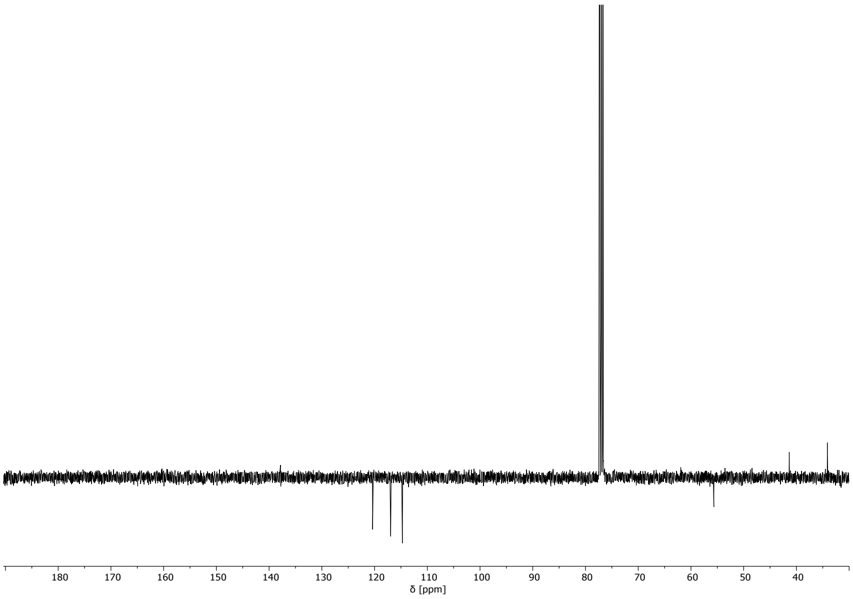

Supplementary Fig. 29. ^{13}C-NMR of compound 3.

Recyclability tests. Recyclability screening was conducted for the reaction between iodoanisole and phenol, following the same procedure of Ullmann couplings. After reaction completion, 100µL of the crude reaction mixture was dissolved in 1 mL methanol. This solution was submitted to GC/MS, quantifying the product conversion. The residual crude reaction mixture was dissolved in 30mL methanol and submitted to centrifugation (15min, 4800rpm). The solvent was decanted. This procedure was repeated 3 times. The obtained black residue was dried under vacuum for 16h, before starting the next reaction cycle. 4 reaction cycles were conducted in total.

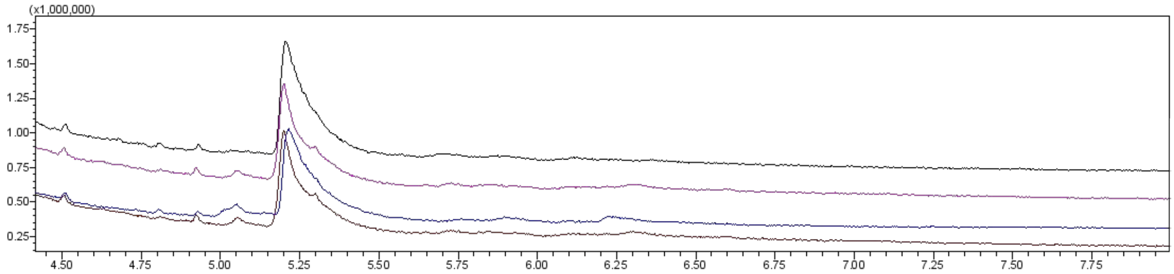

Supplementary Fig. 30. GC/MS chromatograms of the Cu-SAC recyclability test for the reaction of phenol with 4-iodoanisole. Black= 1st cycle; Pink= 2nd cycle; Blue= 3rd cycle; Brown= 4th cycle

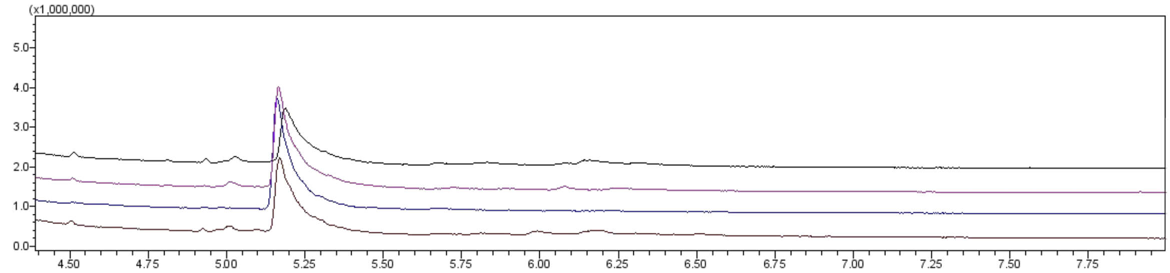

Supplementary Fig. 31. Comparison of the GC/MS chromatograms for all reference compounds. The reaction of 4-iodoanisole with phenol was investigated. Brown= CuI; Blue= Cu_2O; Pink= Cu-$NC^{0.87}$; Black= Cu-NC^{AP};

REFERENCES AND NOTES

(1) R. B. Bird. *Applied Mechanics Reviews*, **2002**, *55*. R1.

(2) M. K. Z. Daniel M. Dobkin. *Principles of Chemical Vapor Deposition,* **2003**

(3) X.-T. Yan and Y. Xu. *Chemical Vapour Deposition* Springer London, **2010.**

(4) Primbs, M. et al. Establishing reactivity descriptors for platinum group metal (PGM)-free Fe-N-C catalysts for PEM fuel cells. *Energ. Environ. Sci.* **13**, 2480-2500 (2020)

(5) Mehmood, A. et al. High loading of single atomic iron sites in Fe-NC oxygen reduction catalysts for proton exchange membrane fuel cells. *Nat. Catal.* **5**, 311-323 (2022).
(6) Jiao, L. et al. Chemical vapour deposition of Fe-N-C oxygen reduction catalysts with full utilization of dense Fe-N sites. *Nat. Mater.* **20**, 1385 (2021).
